# A multi-subgroup predictive model based on clinical parameters and laboratory biomarkers to predict in-hospital outcomes of plasma exchange-centered artificial liver treatment in patients with hepatitis B virus-related acute-on-chronic liver failure

**DOI:** 10.3389/fcimb.2023.1107351

**Published:** 2023-03-21

**Authors:** Jie Liu, Xinrong Shi, Hongmin Xu, Yaqiong Tian, Chaoyi Ren, Jianbiao Li, Shigang Shan, Shuye Liu

**Affiliations:** ^1^Clinical Laboratory Department, The Third Central Hospital of Tianjin, Tianjin, China; ^2^Tianjin Key Laboratory of Extracorporeal Life Support for Critical Diseases, Tianjin, China; ^3^Artificial Cell Engineering Technology Research Center, Tianjin, China; ^4^Tianjin Institute of Hepatobiliary Disease, Tianjin, China; ^5^Hepatobiliary Surgery Department, The Third Central Hospital of Tianjin, Tianjin, China

**Keywords:** hepatitis B virus-related acute-on-chronic liver failure, artificial liver support system, prognosis, plasma exchange, calibration, discrimination

## Abstract

**Background:**

Postoperative risk stratification is challenging in patients with hepatitis B virus-related acute-on-chronic liver failure (HBV-ACLF) who undergo artificial liver treatment. This study characterizes patients’ clinical parameters and laboratory biomarkers with different in-hospital outcomes. The purpose was to establish a multi-subgroup combined predictive model and analyze its predictive capability.

**Methods:**

We enrolled HBV-ACLF patients who received plasma exchange (PE)-centered artificial liver support system (ALSS) therapy from May 6, 2017, to April 6, 2022. There were 110 patients who died (the death group) and 110 propensity score-matched patients who achieved satisfactory outcomes (the survivor group). We compared baseline, before ALSS, after ALSS, and change ratios of laboratory biomarkers. Outcome prediction models were established by generalized estimating equations (GEE). The discrimination was assessed using receiver operating characteristic analyses. Calibration plots compared the mean predicted probability and the mean observed outcome.

**Results:**

We built a multi-subgroup predictive model (at admission; before ALSS; after ALSS; change ratio) to predict in-hospital outcomes of HBV-ACLF patients who received PE-centered ALSS. There were 110 patients with 363 ALSS sessions who survived and 110 who did not, and 363 ALSS sessions were analyzed. The univariate GEE models revealed that several parameters were independent risk factors. Clinical parameters and laboratory biomarkers were entered into the multivariate GEE model. The discriminative power of the multivariate GEE models was excellent, and calibration showed better agreement between the predicted and observed probabilities than the univariate models.

**Conclusions:**

The multi-subgroup combined predictive model generated accurate prognostic information for patients undergoing HBV-ACLF patients who received PE-centered ALSS.

## 1 Introduction

Acute-on-chronic liver failure (ACLF) is a complex syndrome that results in a short-term mortality rate of 50%–90% (Wu et al., 2018; Zaccherini et al., 2020; Li et al., 2021). In China, patients with hepatitis B virus-related ACLF (HBV-ACLF) account for 70% of the total (Gu et al., 2018). The mortality rate is high, and treatment is challenging (Chan and Fan, 2015; Artru et al., 2017). Treatment of HBV-ACLF includes general management, specific treatment, bridging therapies, and liver transplantation. General management and antiviral strategies might improve outcomes; however, this suggestion is controversial (Garg et al., 2011; Luo et al., 2023). Liver transplantation (LT) is the most effective therapy, with a one-year survival rate of 87% (Finkenstedt et al., 2013; Chan and Fan, 2015); however, the difficulties associated with urgent transplantation assessment and the lack of donors limit its application.

An ALSS can serve as an alternative treatment for liver failure; it preserves the liver, kidney, and coagulation systems’ functions, reduces the end-stage liver disease (MELD) scores and mortality model, and prolongs the waiting time for a liver donor. The outcomes of ALSS for HBV-ACLF are challenging to predict (Alshamsi et al., 2020), resulting in multiple ALSS treatments, excessive plasma consumption, and high costs.

Patients with HBV-ACLF generally require three to five ALSS procedures; some even require more than ten procedures. Ignoring correlations of several treatments for the same patient might reduce the efficiency of the estimation and generate significant false positive rates when the correlation is substantial. It is essential to consider correlations when analyzing operation sessions (Niu et al., 2020). Furthermore, no predictive models can reassess patients’ in-hospital outcomes before/after each operation session of ALSS treatment. Existing models predict outcomes within a relatively short or long time; none target in-hospital outcomes. Therefore, a predictive model is needed to maximize the therapeutic value of ALSS treatment and assist in timely decision-making regarding continuing ALSS or preparation for LT.

This study was designed to generate a multi-subgroup combined predictive model based on clinical parameters and laboratory biomarkers to predict in-hospital outcomes of plasma exchange (PE)-centered artificial liver treatment in patients with HBV- ACLF. Based on the characteristics of multiple artificial liver treatments during the hospital, generalized estimating equations (GEE) results are expected. The GEE model estimates binary outcomes (survival in-hospital, yes/no) when clustered data are used (several sessions of ALSS in one patient). All clinical parameters and laboratory biomarkers were divided into subgroups according to time during hospitalization to generate predictive models that can reassess patients’ in-hospital outcomes before/after each operation session of ALSS treatment. Our new -subgroup combined predictive model was derived from an HBV-ACLF clinical database, including the most common clinical management indicators.

## 2 Materials and methods

### 2.1 Participant selection

Patients diagnosed with ACLF, as proposed by the Asian Pacific Association for the Study of the Liver (Sarin et al., 2019), were recruited from the general clinic population from May 6, 2017, to April 6, 2022. Inclusion criteria were as follows: (a) 18–80 years of age; (b) chronic hepatitis B infections; (c) PE or PE+ double plasma molecular absorb system (DPMAS) or PE+ hemofiltration ALSS support; (d) no medical history of LT. Exclusion criteria were as follows: (a) hepatocellular carcinoma, severe extrahepatic diseases, or other tumors; (b) cardiopulmonary disease or bleeding; (c) hypersensitivity reactions to plasma, human albumin, or heparin. This study also included patients who presented without ACLF at admission but later developed ACLF before starting ALSS therapy.

Many artificial liver treatment modes are often used in combination with clinical practice. To achieve the purpose of this study while complying with real-world evidence, we enrolled HBV-ACLF patients who received PE-centered ALSS therapy (PE alone or in combination with hemofiltration, DPMAS, or plasma perfusion). On the other hand, there were often multiple ALSS procedures in one patient. Under these circumstances, we used propensity score-match (PSM) to eliminate selection bias. PSM between the death group and the survivor group was developed according to the estimated propensity scores, which were calculated using a logistic regression model for the existence of ALSS as a function of the following parameters: sex, number of ALSS sessions, and modes of ALSS sessions. These confounding factors can be a primary source of heterogeneity in predicting in-hospital outcomes (Deidda et al., 2019). The matching was performed using nearest-neighbor matching within 0.2 standard deviations of pooled propensity scores.

### 2.2 Ethics statement

All procedures involving human participants accorded with the ethical standards of the institutional and national research committee and with the 2013 Declaration of Helsinki and its later amendments or comparable ethical standards. The Bioethics Committee of the Third Central Hospital of Tianjin approved the study. Appropriate approvals were obtained from patients or their legal surrogates before enrollment.

### 2.3 Treatment

During hospitalization, all patients received standard medical therapy. Complications were also treated (Xie et al., 2020), including a high-calorie diet, nucleoside analogs for HBV DNA-positive patients, sodium restriction (when necessary), diuretics and paracentesis with albumin infusion for ascites, and prophylactic antibiotics for bacterial infections. After the patient’s condition was stabilized, ALSS was performed every two to three times per week and was discontinued if bleeding or circulatory complications occurred (Chen et al., 2016).

### 2.4 Data collection

Coagulation assays prothrombin activity (PTA), international normalized ratio (INR), activated partial thromboplastin time (APTT), fibrinogen (FBG), thrombin time(TT), antithrombin (AT) and fibrinogen degradation products (FDP) were performed using a Stago automated coagulation analyzer (STA Compact Max^®^, France) according to the manufacturer’s instructions. D-dimer was measured using a fluorometric method on a GP1600 (Getein Biotech). A Roche Cobas 8000 c701 automated chemistry analyzer (Roche Diagnostics, Mannheim, Germany) was used to measure total protein (TP), albumin (ALB), globulin (Glb), total bilirubin (TBil), direct bilirubin (DBil), indirect bilirubin (IBil), alanine aminotransferase (ALT), aspartate aminotransferase (AST), mitochondrial aspartate transaminase (m-AST), alkaline phosphatase (ALP), gamma-glutamyl transpeptidase (GGT), total bile acid (TBA), cholinesterase (CHE), prealbumin (PA), creatinine (CR), Na, Urea and uric acid (UA). Neutrophils (NEUT), neutrophil-to-lymphocyte ratio (NLR), platelet-to-lymphocyte ratio (PLR) and platelets (PLT) were measured using the Advia 2120 hematology system (Siemens).

Data were collected according to case report forms. All clinical parameters and laboratory biomarkers were divided into four subgroups (baseline; before ALSS; after ALSS; change ratio) according to the patient’s state. Parameters were measured early in the day after admission to generate the baseline group. Laboratory biomarkers tested in the early morning before each session of ALSS treatment were the before-ALSS group; those tested after each ALSS treatment were the after-ALSS group. The change ratio = (post-treatment - pre-treatment level)/pre-treatment. The predictive models (baseline, pre-model, post-model, change model) were built using the data in each subgroup according to the in-hospital outcomes.

### 2.5 Statistical analysis

IBM SPSS Statistics for Windows software package, version 25 (IBM Corp., Armonk, N.Y., USA) and GraphPad Prism version 9.3.1 (GraphPad Software, Inc., San Diego, California) were used for statistical analysis. Metric data were expressed as mean values with standard deviation or median (p25, p75). The qualitative data were expressed as frequency and composition. Because ALSS sessions for a single patient might correlate with one another more closely than the sessions between patients, GEE models were used to manage the clustering. Univariate and multivariate GEE analyses were performed. A forward stepwise selection method was used to select variables for the multivariate GEE model. A quasi-likelihood under the independence model criterion (QIC) was calculated; the model with the smallest QIC was the most parsimonious (Pan, 2001). The standardized regression coefficient β was calculated as a tracking coefficient (Twisk et al., 1996). Discrimination and calibration are critical aspects characterizing the performance of a prediction model (Steyerberg et al., 2010; Moons et al., 2015). Calibration and discrimination were performed to determine whether the GEE models were adequate for their purpose. The discrimination was assessed by comparing the receiver operating characteristic (ROC) curves using the z-test (Delong’s method (DeLong et al., 1988). The discriminative power is excellent if the area under the ROC curve is > 0.80, very good if > 0.75, and good (acceptable) if > 0.70 (Moons et al., 2015). Using GraphPad Prism software, calibration plots were used to compare the mean predicted probability and the mean observed outcome. Results were displayed graphically with predicted outcome probabilities (Y-axis) plotted against observed outcome frequencies (X-axis). The goodness-of-fit test measures the differences between observed and expected outcomes over deciles (ten groups of patients) of risk (Moons et al., 2015; Nezic, 2020). Perfect predictions should be on the 45° line (Steyerberg et al., 2010). A P-value < 0.05 was considered statistically significant.

## 3 Results

### 3.1 Study population

The design is summarized in **Figure S1**. We included 110 consecutive patients who died in the hospital (death group) and 110 PSM patients who survived to discharge (survivor group). After PSM, in the death or survivor groups, 22 patients underwent one session of PE-centered ALSS treatment; 18 underwent two; 26 underwent three; 11 underwent four; 15 underwent five; 18 underwent six sessions. There were 726 sessions of PE-centered ALSS treatment for 220 patients, with an average of 3.3 sessions per patient (1–6 sessions per patient).

### 3.2 Clinical parameters and laboratory biomarkers at admission associated with outcomes

One hundred ten patients survived in-hospital, and 110 did not. A comparison of baseline characteristics between the death and survivor group density is displayed in **Table 1**. As shown in the table, HBV-ACLF patients in the survivor group were significantly younger and had substantially lower levels of pulse (*P*=0.001), INR (*P*<0.0001), APTT(*P*<0.0001), D-dimer (*P*=0.001), TBil (*P*=0.02), DBil (*P*=0.04), IBil (*P*=0.025), CR (*P*=0.022), MELD scores (*P*=0.002), and 3-month mortality based on MELD scores (*P*=0.001) than non-survivors; they also had higher levels of PTA (*P*<0.0001), ALT (*P*<0.0001), AST (*P*=0.008), m-AST (*P*=0.006), and CHE (*P*<0.0001) at admission than non-survivors.

**Table 1:** Comparison of baseline characteristics between the death and survivor groups density.

Parameters	Survival in-hospital		Wald Chi-Square	*P*	QIC
	YES	NO			
	(N=110)	(N=110)			
Sex (Male/Female)	71/39	59/51	2.70	0.100	306.27
Age (year)	52.80 ± 12.82	61.27 ± 9.75	22.01	<0.0001	280.22
Pulse (/min)	78.00(75.00,83.00)	86.00(78.00,95.25)	11.48	0.001	290.64
SYS (mmHg)	122.51 ± 10.35	118.41 ± 18.73	1.71	0.190	128.59
DIA (mmHg)	74.21 ± 8.61	71.24 ± 13.20	1.53	0.217	128.82
PTA (%)	46.50(40.00,56.00)	40.00(33.00,52.00)	18.22	<0.0001	291.88
INR	1.71(1.47,1.95)	1.93(1.56,2.27)	17.71	<0.0001	288.95
APTT (S)	41.55(38.03,46.03)	46.35(41.45,53.03)	18.28	<0.0001	279.17
FBG (s)	17.60(14.20,25.60)	18.05(14.60,23.23)	0.13	0.722	308.88
TT (s)	19.90(17.98,21.53)	19.40(17.80,21.98)	1.36	0.244	307.07
AT (%)	38.00(30.00,50.00)	33.00(21.00,55.00)	0.50	0.482	216.08
D-dimer (mg/L,DDU)	0.61(0.24,1.63)	1.99(1.10,3.05)	11.40	0.001	284.96
FDP (mg/L)	3.40(1.70,7.55)	5.60(3.00,8.70)	0.54	0.464	207.07
TP (g/L)	56.65(51.55,62.75)	55.60(50.90,60.70)	2.50	0.114	306.45
ALB (g/L)	32.20(29.48,35.85)	33.30(30.3,36.30)	0.24	0.623	304.58
Glb (g/L)	24.50(19.73,26.93)	22.10(18.70,26.80)	3.00	0.083	301.72
A/G	1.40(1.07,1.70)	1.48(1.22,1.86)	0.00	0.975	304.66
TBil (umol/L)	291.05(206.63,355.60)	326.80(223.63,424.95)	5.45	0.020	303.29
DBil (umol/L)	200.19 ± 83.81	225.69 ± 95.03	4.24	0.040	304.58
IBil (umol/L)	77.15(62.65,101.45)	92.60(61.78,125.30)	5.00	0.025	303.86
DBil/TBil	0.72(0.65,0.77)	0.72(0.64,0.76)	0.00	0.956	308.98
ALT (U/L)	239.50(95.00,566.25)	122.00(58.50,252.00)	17.21	<0.0001	288.30
AST (U/L)	253.50(159.50,523.25)	180.50(92.50,408.00)	6.95	0.008	301.61
m-AST (U/L)	26.50(18.08,62.65)	18.95(11.28,38.60)	7.48	0.006	299.06
m-AST/AST	0.10(0.08,0.15)	0.11(0.08,0.14)	2.41	0.121	303.71
ALP (U/L)	154.00(116.75,231.00)	177.00(125.00,235.00)	0.09	0.759	304.70
GGT (U/L)	184.50(107.00,382.75)	187.00(86.00,340.00)	2.60	0.107	300.56
TBA (umol/L)	375.90(263.25,493.35)	332.85(225.40,493.15)	0.02	0.904	307.62
CHE (U/L)	3387.50(2494.25,4673.25)	2918.00(2140.00,3644.25)	12.66	<0.0001	295.92
PA (mg/dl)	5.40(4.10,7.75)	6.05(4.08,8.63)	0.31	0.579	290.73
CR (umol/L)	63.50(54.00,76.50)	65.00(52.75,86.25)	5.27	0.022	303.94
Na (mmol/L)	136.10(133.90,138.58)	136.20(132.80,138.80)	0.36	0.548	296.12
Urea (mmol/L)	4.89(3.31,6.77)	5.20(3.53,7.17)	0.17	0.678	296.14
UA (umol/L)	180.00(119.00,250.00)	165.00(130.25,248.00)	1.08	0.298	275.43
NEUT (%)	72.00(63.78,79.95)	74.50(66.53,81.98)	2.20	0.138	219.10
NLR	4.38(2.66,7.78)	4.96(3.13,10.38)	0.08	0.780	215.79
PLR	125.71(87.56,188.19)	123.88(85.98,182.89)	0.23	0.629	214.29
PLT (10^9^/L)	133.00(80.50,180.00)	101.00(58.50,163.50)	2.13	0.144	212.54
Meld score	24.00(22.00,26.00)	26.00(23.00,29.00)	9.87	0.002	296.33
3-Month Mortality (%)	19.60(19.60,19.60)	19.60(19.60,52.60)	10.17	0.001	297.95

QIC, Quasi likelihood under the independence model criterion; SYS, systolic pressure; DIA, diastolic pressure; PTA, prothrombin time activity; INR, international normalized ratio; APTT, activated partial thromboplastin time; FBG, fibrinogen; TT, thrombin time; AT, antithrombin; FDP, fibrinogen degradation products; TP, total protein; ALB albumin; Glb, globulin; TBil, total bilirubin; DBIL, direct bilirubin; IBil, indirect bilirubin; ALT, alanine aminotransferase; AST, aspartate aminotransferase; m-AST, mitochondrial aspartate transaminase; ALP, alkaline phosphatase; GGT, gamma-glutamyl transpeptidase; TBA, total bile acid; CHE, cholinesterase; PA, prealbumin; CR, creatinine; UA, uric acid; NEUT, neutrophils; NLR, neutrophil-to-lymphocyte ratio; PLR, platelet-to-lymphocyte ratio; PLT, platelets; MELD, model for end stage liver disease; 3-Month Mortality, 3-month mortality based on MELD scores.

APTT(*P*<0.0001), Age (*P*<0.0001), D-dimer (*P*=0.011), ALT(*P*<0.0001), pulse (*P*=0.001), and TBil (*P*=0.016) were entered into a multivariate GEE model (**Table 2**), which was the most parsimonious and had the smallest QIC (211.55).

**Table 2:** Multivariate GEE model for the effect of sessions of ALSS therapy on prognosis of patients with HBV-ACLF.

Predictors	β	OR (95% CI)	*P*	QIC	AUC (95% CI)	Cut-off	Sensitivity	Specificity
At admission								
APTT (S)	-0.075	0.928(0.890,0.967)	<0.0001	211.55	0.872(0.826,0.918)^a^	0.67^b^	0.68	0.93
Age (year)	-0.072	0.930(0.899,0.963)	<0.0001					
D-dimer (mg/L,DDU)	-0.297	0.743(0.591,0.933)	0.011					
ALT (U/L)	0.003	0.997(0.996,0.998)	<0.0001					
Pulse (/min)	-0.064	0.938(0.903,0.973)	0.001					
TBil (umol/L)	-0.004	0.996(0.993,0.999)	0.016					
Before ALSS treatment								
TBil (umol/L)	-0.012	0.988(0.986,0.991)	<0.0001	622.11	0.885(0.862,0.909)^a^	0.60^b^	0.76	0.84
Glb (g/L)	0.100	1.105(1.057,1.156)	<0.0001					
APTT (s)	-0.063	0.939(0.910,0.968)	0.010					
GGT (U/L)	0.002	1.002(1.001,1.003)	0.003					
UA (umol/L)	-0.005	0.996(0.993,0.998)	<0.0001					
After ALSS treatment								
Meld score	-0.249	0.780(0.707,0.860)	<0.0001	530.88	0.888(0.862,0.914)^a^	0.47^b^	0.87	0.80
TBil (umol/L)	-0.011	0.989(0.984,0.993)	<0.0001					
Glb (g/L)	0.180	1.197(1.130,1.269)	<0.0001					
PA (mg/dl)	0.134	1.144(1.061,1.233)	<0.0001					
UA (umol/L)	-0.005	0.995(0.992,0.997)	<0.0001					
Change ratio (Post-Pre)/Pre								
Meld score	-4.163	0.016(0.004,0.059)	<0.0001	843.09	0.779(0.746,0.813)^a^	0.45^b^	0.83	0.61
CR (umol/L)	-0.893	0.409(0.268,0.626)	<0.0001					
TP (g/L)	3.753	42.657(13.093,138.976)	<0.0001					
AST (U/L)	-0.453	0.636(0.429,0.943)	0.024					
m-AST/AST	-0.426	0.653(0.528,0.808)	<0.0001					
D-dimer (mg/L,DDU)	-0.092	0.912(0.847,0.983)	0.015					
NEUT (%)	-0.705	0.494(0.251,0.972)	0.041					
DBil/TBil	-1.607	0.200(0.056,0.722)	0.014					

OR, odds ratio; QIC, Quasi likelihood under the independence model criterion; AUC, area under the curve; APTT, activated partial thromboplastin time; ALT, alanine aminotransferase; TBil, total bilirubin; Glb, globulin; GGT, gamma-glutamyl transpeptidase; UA, uric acid; MELD, model for end stage liver disease; PA, prealbumin; CR, creatinine; TP, total protein; AST, aspartate aminotransferase; NEUT, neutrophils; DBIL, direct bilirubin.

^a^ The difference of AUC between the multivariate model and the univariate models was statistically significant (P<0.001).

^b^ The value of predicted probability which was calculated on the basis of the multivariate GEE model was set as the cut-off value.

**Figure 1:**
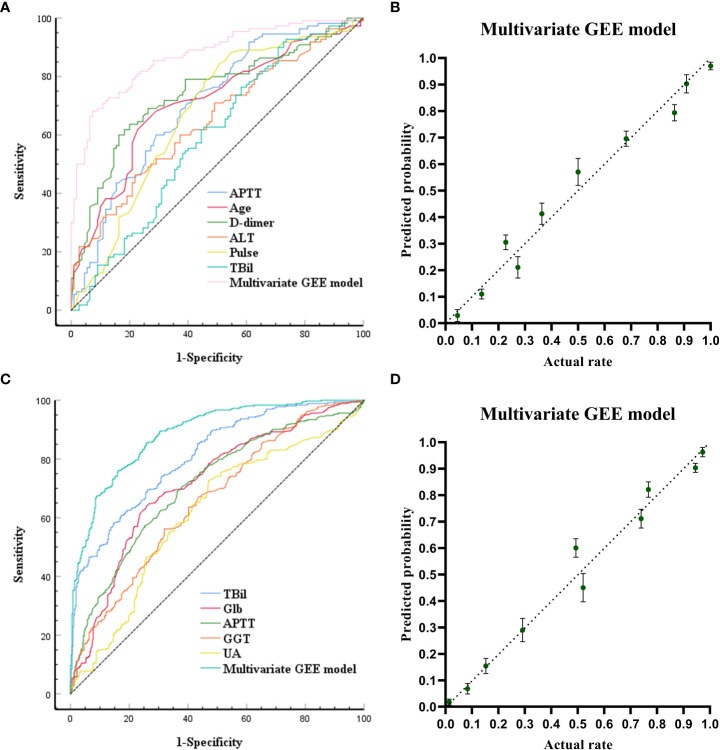
Receiving Operating Characteristic Curve and calibration plots for the baseline models (models built at the time of admission) and the pre-models (models built before ALSS treatment) to predict in-hospital outcomes. **(A)** ROC curve analyses for the capability of APTT, AGE, D-dimer, ALT, pulse, TB at admission and the multivariate generalized estimating equation (GEE) model to predict in-hospital outcomes. **(B)** Calibration plots for the baseline multivariate models to predict in-hospital outcomes. **(C)** ROC curve analyses for the capability of TB, globulin, APTT, GGT, UA before ALSS and the multivariate generalized estimating equation (GEE) model to predict in-hospital outcomes. **(D)** Calibration plots for the pre- multivariate models to predict in-hospital outcomes.

The ROC curves and calibration plots are shown in **Figure 1**. The discriminative power of the multivariate GEE model was excellent (AUC=0.872) (**Figure 1A**), better than APTT (AUC=0.707), Age (AUC=0.711), D-dimer (AUC=0.742), ALT(AUC=0.650), pulse(AUC=0.672) or TBil (AUC=0.593) alone (**Table S1**). Calibration of the multivariate model (Y = 0.9653*X + 0.01733, (R2 = 0.9677, *P*<0.0001)) (**Figure 1B**) showed better agreement between the predicted and observed probabilities than the univariate models (**Figure S2**).

### 3.3 Analysis of laboratory biomarkers associated with outcomes before ALSS treatment

The laboratory biomarkers before ALSS treatment were compared between the 363 survivors and 363 non-survivors. Relationships between laboratory biomarkers before ALSS treatment and in-hospital outcomes is shown in **Table S2**, HBV-ACLF patients in the survivor group had substantially lower levels of INR (*P*=0.006), APTT(*P*<0.0001), D-dimer (*P*=0.006), TBil (*P*<0.0001), DBil (*P*<0.0001), IBil (*P*<0.0001), DBil/TBil (*P*=0.001), CR (*P*=0.032), Urea (*P*=0.004), UA (*P*=0.002), NEUT (*P*=0.007), MELD Sore (*P*<0.0001), and 3-month mortality based on MELD scores (*P*<0.0001), but had higher levels of PTA (*P*<0.0001), AT (*P*=0.004), TP (*P*<0.0001), Glb (*P*<0.0001), GGT (*P*=0.004), CHE (*P*<0.0001), PA (*P*<0.0001), Na (*P*=0.006) and PLT (*P*<0.0001) before ALSS treatment than patients who did not survive.

TBil (*P*<0.0001), Glb (*P*<0.0001), APTT (*P*=0.01), GGT(*P*=0.003) and UA (*P*<0.0001) were entered in multivariate GEE model (**Table 2**), which was the most parsimonious with the smallest QIC (622.11).

The ROC curves and calibration plots are shown in **Figure 1**. The discriminative power of the multivariate GEE model was excellent (AUC=0.885) (**Figure 1C**), better than TBil (AUC=0.808), Glb (AUC=0.716), APTT (AUC=0.709), GGT(AUC=0.658), or UA (AUC=0.614) alone (**Table S3**). Calibration of the multivariate model (Y = 0.9878*X + 0.006093 (R2 = 0.9715, *P*<0.0001)) (**Figure 1D**) showed better agreement between the predicted and observed probabilities than the univariate models (**Figure S3**).

### 3.4 Laboratory biomarkers associated with outcomes after ALSS treatment

The laboratory biomarkers after ALSS treatment were compared between the 363 survivors and 363 non-survivors. Relationships between laboratory biomarkers after ALSS treatment and in-hospital outcomes is shown in **Table S4**, HBV-ACLF patients in the survivor group had substantially lower levels of INR (*P*<0.0001), APTT(*P*=0.001), D-dimer (*P*=0.017), TBil (*P*<0.0001), DBil (*P*<0.0001), IBil (*P*<0.0001), DBil/TBil (*P*<0.0001), CR (*P*<0.0001), Urea (*P*<0.0001), UA (*P*=0.001), NEUT (*P*<0.0001), NLR (*P*<0.0001), MELD scores (*P*<0.0001), and 3-month mortality based on MELD scores (*P*<0.0001), but had higher levels of PTA (*P*=0.001), TP (*P*<0.0001), Glb (*P*<0.0001), GGT (*P*=0.007), PA (*P*<0.0001), Na (*P*<0.0001) and PLT (*P*<0.0001) after ALSS treatment than HBV-ACLF patients who did not survive.

MELD Sore (*P*<0.0001), TBil (*P*<0.0001), Glb (*P*<0.0001), PA (*P*<0.0001) and UA (*P*<0.0001) were entered into multivariate GEE model (**Table 2**), which is the most parsimonious with the smallest QIC (530.88).

**Figure 2:**
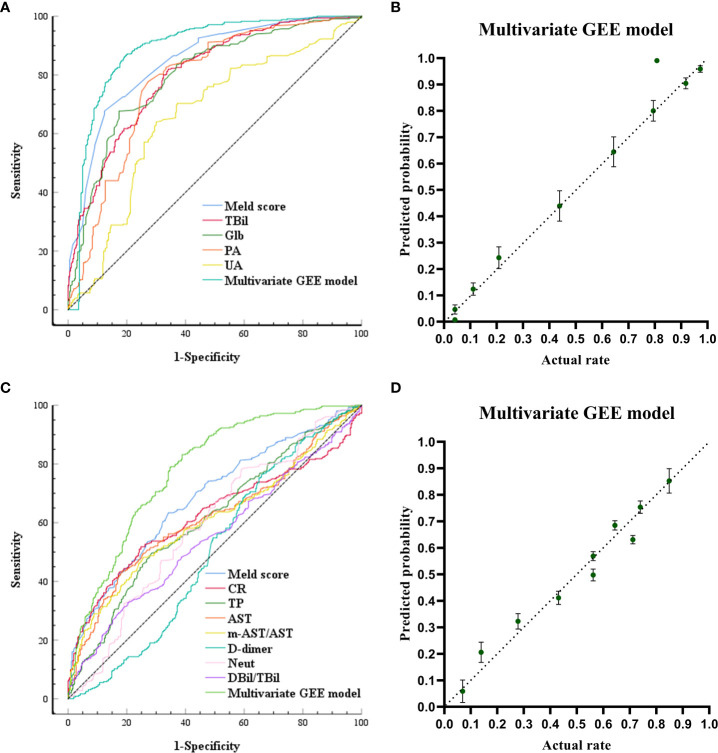
Receiving Operating Characteristic Curve and calibration plots for the post-models (models built after ALSS treatment) and change-models (models built by calculating the rate of change of each laboratory biomarker before and after ALSS treatment: Rate of change = (Post-ALSS-Pre-ALSS)/Pre-ALSS) to predict in-hospital outcomes. **(A)** ROC curve analyses for the capability of Meld, TB, globulin, PA, UA after ALSS and the multivariate generalized estimating equation (GEE) model to predict in-hospital outcomes. **(B)** Calibration plots for the post-multivariate models to predict in-hospital outcomes. **(C)** ROC curve analyses for the capability of change ratio of Meld, CR, TP, AST, m-AST/AST, D-dimer, NEUT, DBIL/TBIL and the multivariate generalized estimating equation (GEE) model to predict in-hospital outcomes. **(D)** Calibration plots for the change-multivariate models to predict in-hospital outcomes.

The ROC curves and calibration plot are shown in **Figure 2**. The discriminative power of the multivariate GEE model was excellent (AUC=0.888) (**Figure 2A**), better than the MELD Sore (AUC=0.851), TBil (AUC=0.803), Glb (AUC=0.797), PA (AUC=0.784) or UA (AUC=0.659) alone (**Table S5**). Calibration of the multivariate model (Y = 1.036*X + 1.670e-005 (R2 = 0.9693, *P*<0.0001)) (**Figure 2B**) showed better agreement between the predicted and observed probabilities than the univariate models (**Figure S4**).

### 3.5 The change ratio of each laboratory biomarker associated with outcomes

Relationships between the rate of change ((Post-ALSS-Pre-ALSS)/Pre-ALSS) of each laboratory biomarker and in-hospital outcomes are shown in **Table S6**. The change ratio was compared between the 363 survivors and 363 non-survivors, HBV-ACLF patients in the survivor group had substantially lower levels of INR (*P*=0.002), DBil/TBil (*P*=0.036), ALT(*P*<0.0001), AST(*P*=0.002), m-AST(*P*=0.046), m-AST/AST(*P*<0.0001), GGT(*P*=0.001), CR (*P*<0.0001), Urea (*P*<0.0001), NEUT (*P*=0.007), MELD scores (*P*<0.0001), and 3-month mortality based on MELD scores (*P*<0.0001), but had higher levels of PTA (*P*=0.040), D-dimer (*P*=0.013), ALB (*P*<0.0001), TP (*P*<0.0001), Glb (*P*<0.0001) and PLT (*P*=0.039) than HBV-ACLF patients who did not survive.

MELD Sore (*P*<0.0001), CR (*P*<0.0001), TP (*P*<0.0001), AST (*P*=0.024), m-AST/AST(*P*<0.0001), D-dimer (*P*=0.015), NEUT (*P*=0.041) and DBil/TBil (*P*=0.014) were entered into multivariate GEE model (**Table 2**), which is the most parsimonious with the smallest QIC (843.09).

The ROC curves and calibration plots are shown in **Figure 2**. The discriminative power of the multivariate GEE model was very good (AUC=0.779) (**Figure 2C**), better than the MELD Sore (AUC=0.683), CR (AUC=0.622), TP (AUC=0.613), AST (AUC=0.617), m-AST/AST (AUC=0.501), D-dimer (AUC=0.503), NEUT (AUC=0.592) or DBil/TBil (AUC=0.550) alone (**Table S7**). Calibration of the multivariate model (Y = 0.9381*X + 0.03090 (R2 = 0.9546, *P*<0.0001)) in **Figure 2D** showed better agreement between the predicted and observed probabilities than the univariate models (**Figure S5**).

## 4 Discussion

This study was designed to identify clinical parameters and laboratory biomarkers signatures to predict in-hospital outcomes of patients with HBV-ACLF. Some studies have previously reported predictive models (Shi et al., 2015; Li et al., 2016; Wu et al., 2018; Xie et al., 2020; Shang et al., 2021). This is the first report in which laboratory biomarkers were divided into subgroups according to time during hospitalization, followed by establishing a multi-subgroup predictive model to obtain accurate prognostic information. Accurate prognostic information is critical for medical decision-making. Accurate prognostic assessment assists patients and physicians in the shared decision-making process, preventing testing in low-risk situations and avoiding delays in treatment when there is a high probability of a favorable net benefit (Alba et al., 2017). Our findings revealed that the GEE model in each subgroup had perfect discriminative power and excellent predictive power for the in-hospital outcomes of patients with HBV-ACLF. Discrimination and calibration are essential for evaluating model performance; however, they remain underreported in the literature (Alba et al., 2017). A systematic review addressing prediction models of cardiovascular outcomes noted that only 63% reported discrimination, and 36% reported calibration (Wessler et al., 2015). In the present study, the discriminative power was perfect (AUC > 0.80), and the predictive power was excellent.

At admission, patients who survived ACLF were characterized by lower APTT, age, D-dimer, pulse, TBil, and higher ALT. Although the correlation between age and outcomes was not found in some studies (Du et al., 2021), our study does find that age is an independent prognostic risk factor for patients with HBV-ACLF, along with other studies (Wu et al., 2018; Xie et al., 2020);. The possible correlation between INR and prognosis was not found in our study, even though INR was reported to be an independent risk factor in predicting the development of HBV-ACLF (Du et al., 2021). In the present study, patients who did not survive ACLF had higher TBil and lower ALT; this finding suggests that when the disease becomes severe, many liver cells die, the liver’s ability to produce ALT is lost, and bilirubin levels rise.

ALSS rapidly removes toxic substances and corrects severe coagulopathies after ALSS treatment. Toxic substances are released because of liver cell necrosis, cholestasis, and bilirubin accumulation in the bile capillary or tissue space (Shang et al., 2021); ALSS provides an internal environment suitable for liver cells to restore liver functions (Saliba et al., 2013). The balance of necrosis and regeneration of liver cells determines outcomes in patients with HBV-ACLF. We found that the change ratio of DBil/TBil reflects the regeneration ability of the liver with the help of ALSS, while the change ratio of m-AST/AST reflects necrosis; this finding explains why the model based on a combination of these parameters had good predictive value.

The MELD score indicated mortality in patients with end-stage liver disease and was used to evaluate the curative effects of ALSS (Xia et al., 2013). Several prognostic models for ACLF showed that an increase in their MELD score characterized patients who did not survive ACLF; however, in survivors, the MELD score decreased (Liu et al., 2022; Vogg et al., 2022). In our study, the MELD score after ALSS and the change ratio of the MELD score were entered into our multivariate GEE model, which had the highest AUC (0.851, 0.683) among the models we used for evaluating prognosis.

Inflammation is central to the development and progression of ACLF, which has been reported previously (Du et al., 2021). The NLR is a systemic marker of subclinical inflammation; an increased ratio predicted outcomes in several disorders (Li et al., 2015; Alkhatip et al., 2021). In the present study, NLR values after ALSS were inversely correlated with in-hospital outcomes. This conclusion agrees with other studies (Fan et al., 2017; Li et al., 2021; Sheng et al., 2022). An NLR > 6.78 after ALSS treatment predicts high mortality risk. NLR could be a fast, easy, and low-cost marker to predict ALSS outcomes in patients with HBV-ACLF. Neutrophils are critical to the non-specific cellular immune system and are at the forefront of the defense against microbial pathogens. Activated neutrophils degranulate and release oxidants that diffuse into hepatocytes and trigger intracellular oxidative stress and mitochondrial dysfunction (Wu et al., 2010). Several studies found that the elevation of neutrophils in ACLF might be correlated with aggravated liver injury (Bhatia et al., 2008; Marques et al., 2012). In the present study, we found that the change ratio of neutrophils was associated with outcomes. The increased proportion of neutrophils after ALSS indicates high mortality risk. Therefore, an increased percentage of neutrophils with a high NLR might increase with the severity of liver damage. Prealbumin is a marker of malnutrition and inflammation. It has been associated with poor prognosis in cardiovascular disease (López et al., 2022), but less is known in HBV-ACLF patients. This study found that lower prealbumin levels after ALSS treatment are independently associated with in-hospital mortality.

The effect of standard medical therapy in the first few days after admission (before the first ALSS treatment) predicted ALSS outcomes. Stabilization of PLT and Glb, increased PA and AT levels, a significant decrease in ALT, AST levels, and recovery of kidney function (Na, Urea, UA) decrease the risk of in-hospital mortality; further studies are required to establish statistical relationships.

This study had several limitations, including a relatively small number of patients who underwent PE-centered ALSS at a single center. Prospective, multicenter studies with a larger sample size are needed to optimize the ability to predict outcomes of ALSS in patients with HBV-ACLF.

## 5 Conclusion

This study built a multi-subgroup combined predictive model, which generated accurate prognostic information for patients undergoing HBV-ACLF patients who received PE-centered ALSS. This model can reassess patients’ in-hospital outcomes before/after each operation session of ALSS treatment and assists patients and physicians in choosing the best treatment plan.

## Data availability statement

The original contributions presented in the study are included in the article/**Supplementary Material**. Further inquiries can be directed to the corresponding author.

## Ethics statement

Written informed consent was obtained from the individual(s) for the publication of any potentially identifiable images or data included in this article.

## Author contributions

JieL, HX and SL designed this study and performed the statistical analysis. JieL and XS did the data curation for eligible studies. YT was major contributor in resources and software. CR, JiaL and SS contributed in methodology and review & editing the manuscript. CR, JieL and SS contributed in methodology and review & editing the manuscript. All authors read and approved the final manuscript. All authors contributed to the article and approved the submitted version.

## References

[B1] Alba A. C. Agoritsas T. Walsh M. Hanna S. Iorio A. Devereaux P. J.. (2017). Discrimination and calibration of clinical prediction models: Users' guides to the medical literature. JAMA 318, 1377–1384. doi: 10.1001/jama.2017.12126 29049590

[B2] Alkhatip AAAMM Kamel M. G. Hamza M. K. Farag E. M. Yassin H. M. Elayashy M.. (2021). The diagnostic and prognostic role of neutrophil-to-lymphocyte ratio in COVID-19: A systematic review and meta-analysis. Expert Rev. Mol. Diagn. 21, 505–514. doi: 10.1080/14737159.2021.1915773 33840351 PMC8074650

[B3] Alshamsi F. Alshammari K. Belley-Cote E. Dionne J. Albrahim T. Albudoor B.. (2020). Extracorporeal liver support in patients with liver failure: A systematic review and meta-analysis of randomized trials. Intensive Care Med. 46, 1–16. doi: 10.1007/s00134-019-05783-y 31588983

[B4] Artru F. Louvet A. Ruiz I. Levesque E. Labreuche J. Ursic-Bedoya J.. (2017). Liver transplantation in the most severely ill cirrhotic patients: A multicenter study in acute-on-chronic liver failure grade 3. J. Hepatol. 67, 708–715. doi: 10.1016/j.jhep.2017.06.009 28645736

[B5] Bhatia V. Bhardwaj P. Elikkottil J. Batra J. Saraya A. (2008). A 7-day profile of oxidative stress and antioxidant status in patients with acute liver failure. Hepatol. Int. 2, 465–470. doi: 10.1007/s12072-008-9098-6 19669321 PMC2716903

[B6] Chan A. C. Fan S. T. (2015). Criteria for liver transplantation in ACLF and outcome. Hepatol. Int. 9, 355–359. doi: 10.1007/s12072-014-9585-x 25788183

[B7] Chen J. J. Huang J. R. Yang Q. Xu X. W. Liu X. L. Hao S. R.. (2016). Plasma exchange-centered artificial liver support system in hepatitis b virus-related acuteon-chronic liver failure: A nationwide prospective multicenter study in China. Hepatobiliary Pancreat Dis. Int. 15, 275–281. doi: 10.1016/s1499-3872(16)60084-x 27298103

[B8] Deidda M. Piras C. Binaghi G. Congia D. Pani A. Boi A.. (2019). Metabolomic fingerprint of coronary blood in STEMI patients depends on the ischemic time and inflammatory state. Sci. Rep. 9, 312. doi: 10.1038/s41598-018-36415-y 30670713 PMC6342950

[B9] DeLong E. R. DeLong D. M. Clarke-Pearson D. L. (1988). Comparing the areas under two or more correlated receiver operating characteristic curves: A nonparametric approach. Biometrics 44, 837–845. 3203132

[B10] Du L. Ma Y. Zhou S. Chen F. Xu Y. Wang M.. (2021). A prognostic score for patients with acute-on-chronic liver failure treated with plasma exchange-centered artificial liver support system. Sci. Rep. 11, 1469. doi: 10.1038/s41598-021-81019-8 33446902 PMC7809456

[B11] Fan Z. EnQiang C. Yao D. L. LiBo Y. Hong L. Lang B.. (2017). Neutrophil-lymphocyte ratio predicts short term mortality in patients with hepatitis b virus-related acute-on-chronic liver failure treated with an artificial liver support system. PLoS One 12, e0175332. doi: 10.1371/journal.pone.0175332 28426800 PMC5398520

[B12] Finkenstedt A. Nachbaur K. Zoller H. Joannidis M. Pratschke J. Graziadei I. W.. (2013). Acute-on-chronic liver failure: excellent outcomes afer liver transplantation but high mortality on the wait list. Liver Transpl. 19, 879–886. doi: 10.1002/lt.2367 23696006

[B13] Garg H. Sarin S. K. Kumar M. Garg V. Sharma B. C. Kumar A. (2011). Tenofovir improves the outcome in patients with spontaneous reactivation of hepatitis b presenting as acute-onchronic liver failure. Hepatology 53, 774–780. doi: 10.1002/hep.24109 21294143

[B14] Gu W. Y. Xu B. Y. Zheng X. Chen J. Wang X. B. Huang Y.. (2018). Acute-on-chronic liver failure in China: Rationale for developing a patient registry and baseline characteristics. Am. J. Epidemiol. 187, 1829–1839. doi: 10.1093/aje/kwy083 29762630

[B15] Li J. Chen Q. Luo X. Hong J. Pan K. Lin X.. (2015). Neutrophil-to-Lymphocyte ratio positively correlates to age in healthy population. J. Clin. Lab. Anal. 29, 437–443. doi: 10.1002/jcla.21791 25277347 PMC6807196

[B16] Li H. Chen L. Y. Zhang N. N. Li S. T. Zeng B. Pavesi M.. (2016). Characteristics, diagnosis and prognosis of acute-onChronic liver failure in cirrhosis associated to hepatitis b. Sci. Rep. 6, 25487. doi: 10.1038/srep25487 27146801 PMC4857102

[B17] Li P. Liang X. Xu S. Xiong Y. Huang J. (2021). A non-bioartificial liver support system combined with transplantation in HBV-related acute-on-chronic liver failure. Sci. Rep. 11, 2975. doi: 10.1038/s41598-021-82719-x 33536531 PMC7859234

[B18] Li J. Liang X. You S. Feng T. Zhou X. Zhu B.. (2021). Development and validation of a new prognostic score for hepatitis b virus-related acute-on-chronic liver failure. J. Hepatol. 75, 1104–1115. doi: 10.1016/j.jhep.2021.05.026 34090929

[B19] Liu W. S. Shen L. J. Tian H. Zhai Q. H. Li D. Z. Song F. J.. (2022). ABC Prognostic classification and MELD 3.0 and COSSH-ACLF II prognostic evaluation in acute-on-chronic liver failure. Zhonghua Gan Zang Bing Za Zhi. 30, 976–980. doi: 10.3760/cma.j.cn501113-20220308-00103 36299192 PMC12770579

[B20] López B. Castañón-Apilánez M. Molina-Gil J. Fernández-Gordón Sánchez S. González G. Reguera Acuña A.. (2022). Serum prealbumin levels on admission as a prognostic marker in stroke patients treated with mechanical thrombectomy. Cerebrovasc Dis. Extra. 12, 103–108. doi: 10.1159/000526354 36007497 PMC9941761

[B21] Luo J. Liang X. Xin J. Li J. Li P. Zhou Q.. (2023). Predicting the Onset of Hepatitis B Virus-Related Acute-on-Chronic Liver Failure. Clin Gastroenterol Hepatol, 21:681–693. doi: 10.1016/j.cgh.2022.03.016 35337983

[B22] Marques P. E. Amaral S. S. Pires D. A. Nogueira L. L. Soriani F. M. Lima B. H.. (2012). Chemokines and mitochondrial products activate neutrophils to amplify organ injury during mouse acute liver failure. Hepatology 56, 1971–1982. doi: 10.1002/hep.25801 22532075

[B23] Moons K. Altman D. Reitsma J. Ioannidis J. Macaskill P. Steyerberg E.. (2015). Transparent reporting of a multivariable prediction model for individual prognosis or diagnosis (TRIPOD): Explanation and elaboration. Ann. Intern. Med. 162, W1–W73. doi: 10.7326/M14-0698 25560730

[B24] Nezic D. G. (2020). Assessing the performance of risk prediction models. Eur. J. Cardiothorac Surg. 58, 401. doi: 10.1093/ejcts/ezaa071 32163550

[B25] Niu Y. Wang X. Cao H. Peng Y. (2020). Variable selection *via* penalized generalized estimating equations for a marginal survival model. Stat. Methods Med. Res. 29, 2493–2506. doi: 10.1177/0962280220901728 31994449

[B26] Pan W. (2001). Akaike's information criterion in generalized estimating equations. Biometrics 57, 120–125. doi: 10.1111/j.0006-341x.2001.00120.x 11252586

[B27] Saliba F. Camus C. Durand F. Mathurin P. Letierce A. Delafosse B.. (2013). Albumin dialysis with a noncell artificial liver support device in patients with acute liver failure: A randomized, controlled trial. Ann. Intern. Med. 159, 522–531. doi: 10.7326/0003-4819-159-8-201310150-00005 24126646

[B28] Sarin S. K. Choudhury A. Sharma M. K. Maiwall R. Al Mahtab M. Rahman S.. (2019). Acute-on-chronic liver failure: Consensus recommendations of the Asian pacific association for the study of the liver (APASL): an update. Hepatol. Int. 13, 353–390. doi: 10.1007/s12072-019-09946-3 31172417 PMC6728300

[B29] Shang J. Wang M. Wen Q. Ma Y. Chen F. Xu Y.. (2021). A novel prognostic model to predict outcome of artificial liver support system treatment. Sci. Rep. 11, 7510. doi: 10.1038/s41598-021-87055-8 33820919 PMC8021558

[B30] Sheng X. Zhou J. Gu X. Wang H. (2022). The effect of artificial liver support system on prognosis of HBV-derived hepatorenal syndrome: A retrospective cohort study. Dis. Markers. 2022, 3451544. doi: 10.1155/2022/3451544 35692884 PMC9177308

[B31] Shi Y. Yang Y. Hu Y. Wu W. Yang Q. Zheng M.. (2015). Acute-on-chronic liver failure precipitated by hepatic injury is distinct from that precipitated by extrahepatic insults. Hepatology 62, 232–242. doi: 10.1002/hep.27795 25800029

[B32] Steyerberg E. W. Vickers A. J. Cook N. R. Gerds T. Gonen M. Obuchowski N.. (2010). Assessing the performance of prediction models: A framework for traditional and novel measures. Epidemiology 21, 128–138. doi: 10.1097/EDE.0b013e3181c30fb2 20010215 PMC3575184

[B33] Twisk J. W. Kemper H. C. Mellenbergh D. J. van Mechelen W. (1996). Factors influencing tracking of cholesterol and high-density lipoprotein: The Amsterdam growth and health study. Prev. Med. 25, 355–364. doi: 10.1006/pmed.1996.0066 8781014

[B34] Vogg J. Maier-Stocker C. Munker S. Mehrl A. Schlosser S. Tews H. C.. (2022). Hepatic perfusion as a new predictor of prognosis and mortality in critical care patients with acute-on-chronic liver failure. Front. Med. (Lausanne). 9. doi: 10.3389/fmed.2022.1008450 PMC9589036 36300192

[B35] Wessler B. S. Lai Yh L. Kramer W. Cangelosi M. Raman G. Lutz J. S.. (2015). Clinical prediction models for cardiovascular disease: Tufts predictive analytics and comparative effectiveness clinical prediction model database. Circ. Cardiovasc. Qual Outcomes. 8, 368–375. doi: 10.1161/CIRCOUTCOMES.115.001693 26152680 PMC4512876

[B36] Wu Z. Han M. Chen T. Yan W. Ning Q. (2010). Acute liver failure: mechanisms of immune-mediated liver injury. Liver Int. 30, 782–794. doi: 10.1111/j.1478-3231.2010.02262.x 20492514

[B37] Wu T. Li J. Shao L. Xin J. Jiang L. Zhou Q.. (2018). Development of diagnostic criteria and a prognostic score for hepatitis b virus-related acute-on-chronic liver failure. Gut 67, 2181–2191. doi: 10.1136/gutjnl-2017-314641 28928275

[B38] Xia Q. Dai X. Zhang Y. Guo Y. Xu X. Yang Q.. (2013). A modified MELD model for Chinese pre-ACLF and ACLF patients and it reveals poor prognosis in pre-ACLF patients. PLoS One 8, e64379. doi: 10.1371/journal.pone.0064379 23755119 PMC3673980

[B39] Xie Z. Violetta L. Chen E. Huang K. Wu D. Xu X.. (2020). A prognostic model for hepatitis b acute-on-chronic liver failure patients treated using a plasma exchange-centered liver support system. J. Clin. Apher. 35, 94–103. doi: 10.1002/jca.21762 31769901 PMC7217207

[B40] Zaccherini G. Weiss E. Moreau R. (2020). Acute-on-chronic liver failure: Definitions, pathophysiology and principles of treatment. JHEP Rep. 3, 100176. doi: 10.1016/j.jhepr.2020.100176 33205036 PMC7652714

