# Supplementary material for: A multi-subgroup predictive model based on clinical parameters and laboratory biomarkers to predict in-hospital outcomes of plasma exchange-centered artificial liver treatment in patients with hepatitis B virus-related acute-on-chronic liver failure
Source: Front Cell Infect Microbiol. 2023 Mar 21;13:1107351. doi: 10.3389/fcimb.2023.1107351 (PMC10072158; doi:10.3389/fcimb.2023.1107351)
Supplement: Supplementary file 1 [file DataSheet_1.docx]

**Supplementary Figure 1.** Flow diagram of the experimental design


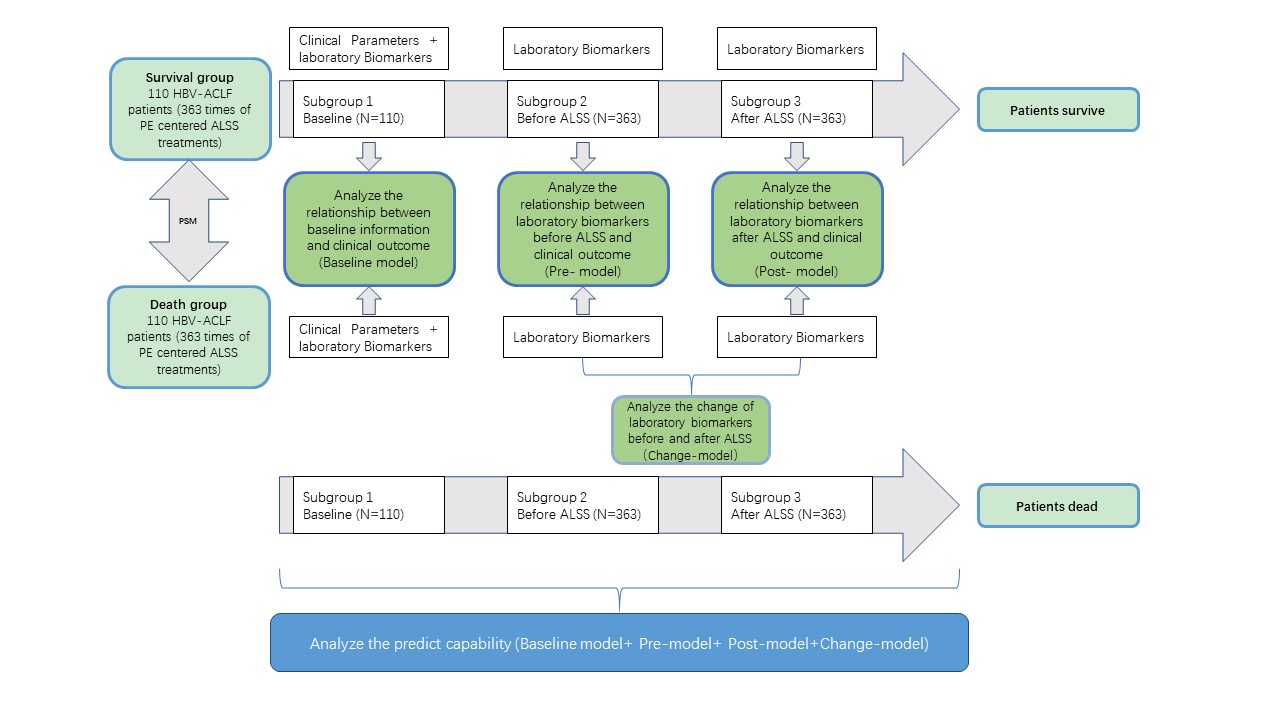


**Supplementary Figure 2.** Calibration of the univariate models at admission


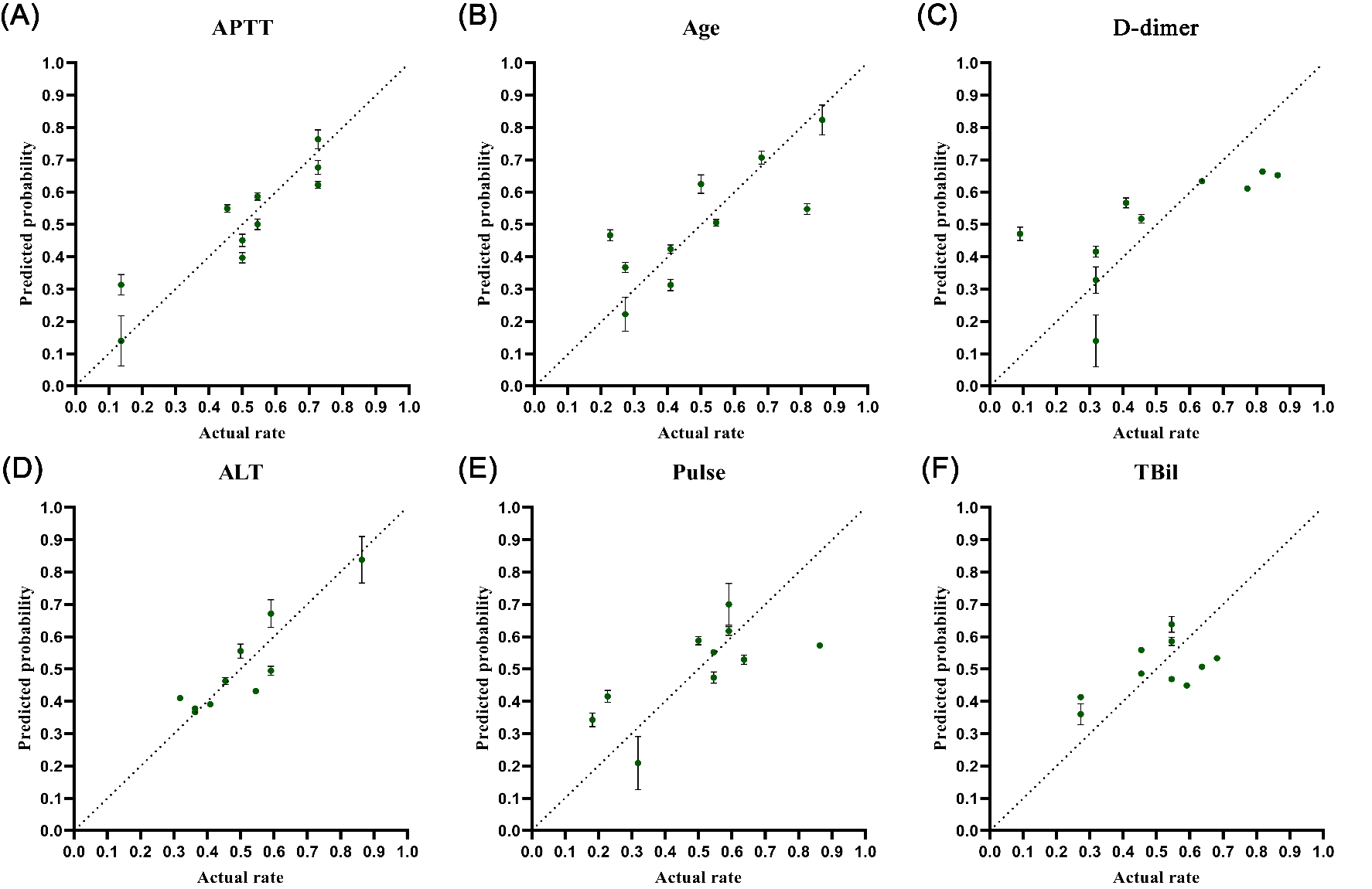


**Supplementary Figure 3.** Calibration of the univariate models before ALSS


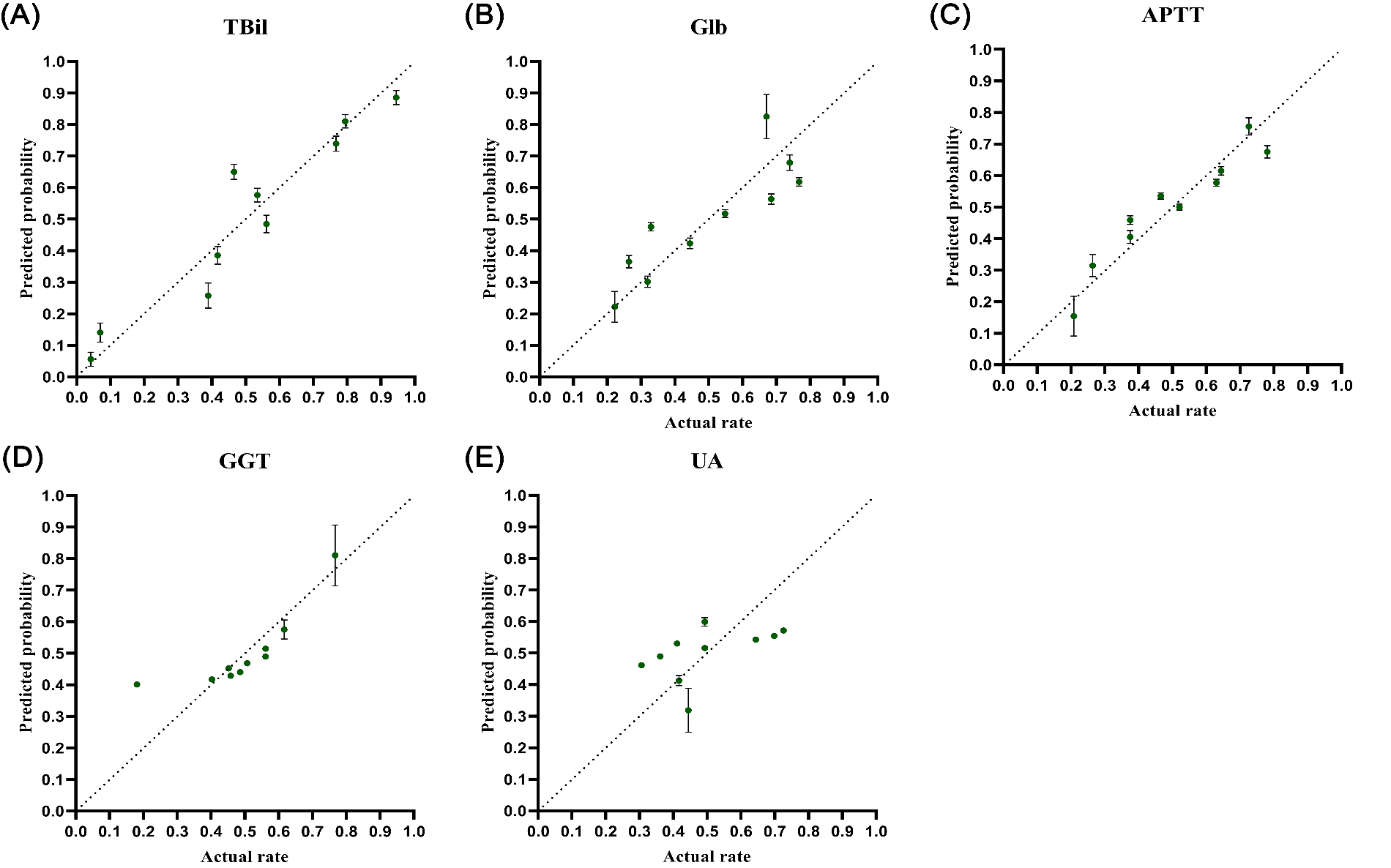


**Supplementary Figure 4.** Calibration of the univariate models after ALSS


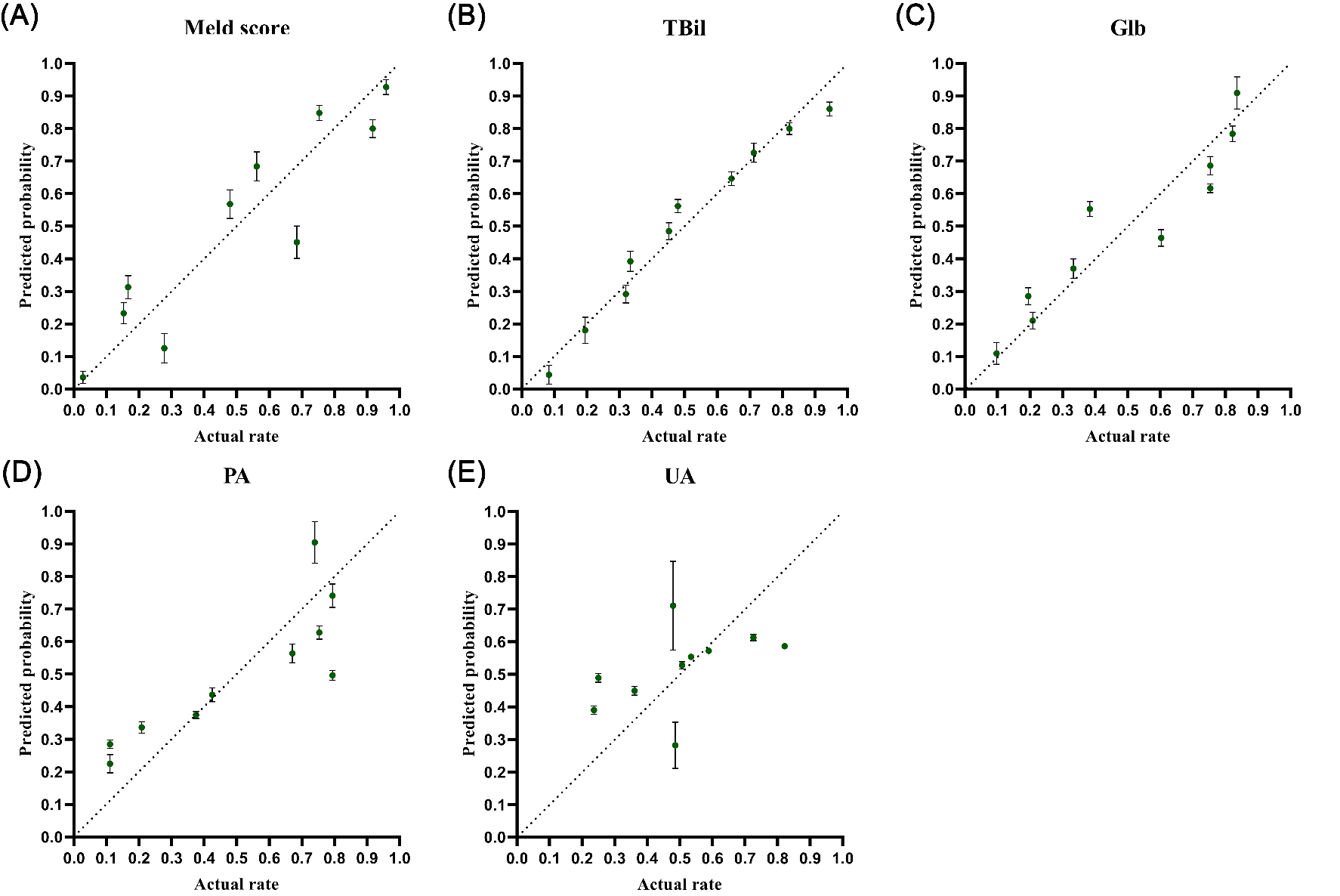


**Supplementary Figure 5.** Calibration of the univariate models of change ratio


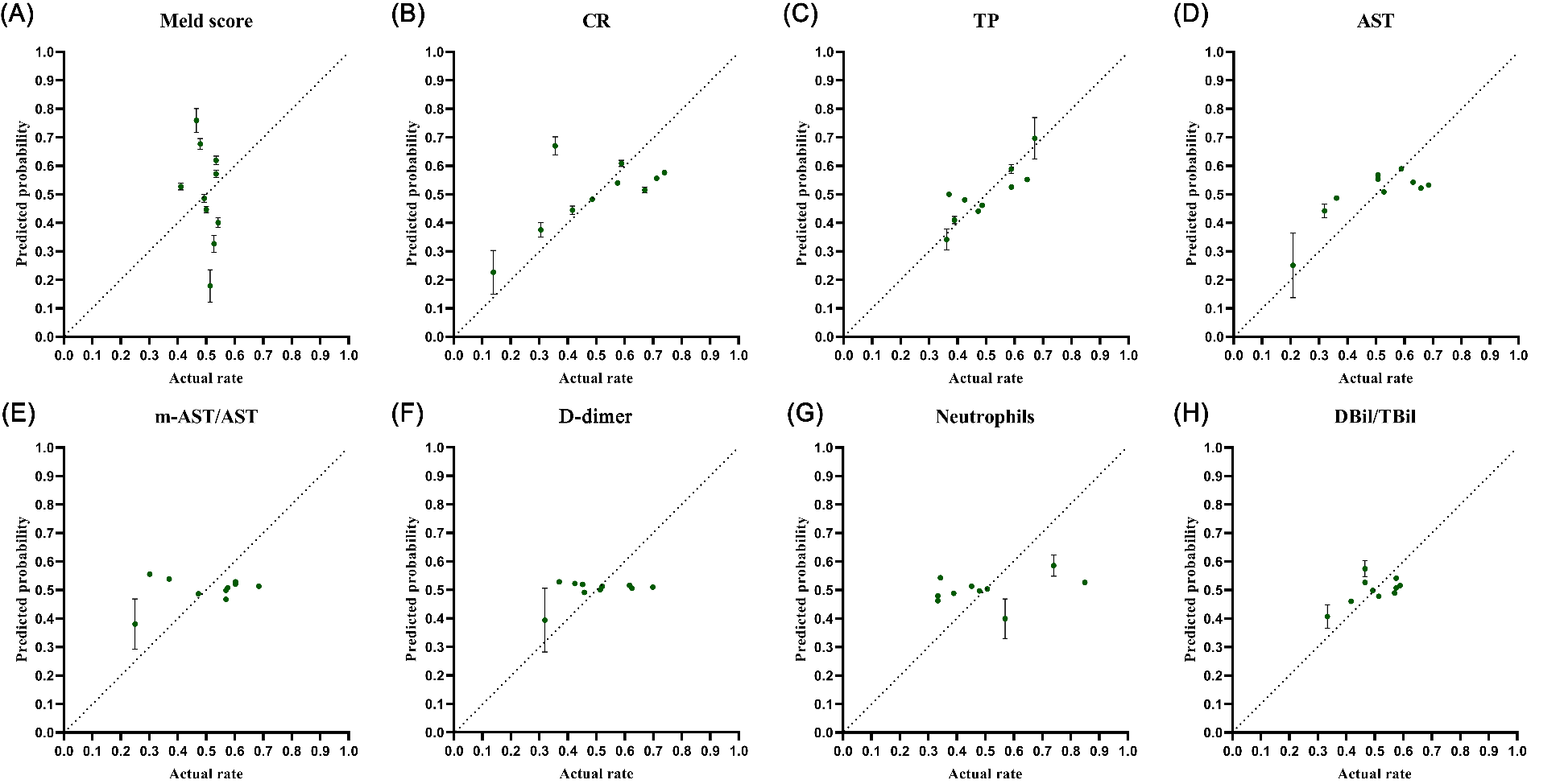


**Supplementary table 1.** Univariate GEE model for patients with HBV-ACLF with full dataset for mortality at admission

| Predictors | OR (95% CI) | *P* | QIC | AUC (95% CI) | Cut-off | Sensitivity | Specificity |
| --- | --- | --- | --- | --- | --- | --- | --- |
| Age (year) | 0.940(0.910,0.960) | <0.0001 | 280.22 | 0.711(0.643,0.78) | 56.50 | 0.77 | 0.62 |
| Pulse (/min) | 0.950(0.921,0.978) | 0.001 | 290.64 | 0.672(0.599,0.744) | 88.50 | 0.46 | 0.88 |
| PTA (%) | 1.045(1.024,1.066) | <0.0001 | 291.88 | 0.639(0.566,0.712) | 39.50 | 0.76 | 0.50 |
| INR | 0.344(0.210,0.566) | <0.0001 | 288.95 | 0.631(0.558,0.705) | 1.89 | 0.54 | 0.73 |
| APTT (S) | 0.911(0.873,0.951) | <0.0001 | 279.17 | 0.707(0.638,0.775) | 49.55 | 0.39 | 0.92 |
| D-dimer (mg/L,DDU) | 0.661(0.52,0.841) | 0.001 | 284.96 | 0.742(0.676,0.808) | 0.95 | 0.80 | 0.64 |
| TBil (umol/L) | 0.997(0.995,1.000) | 0.020 | 303.29 | 0.593(0.518,0.669) | 423.35 | 0.26 | 0.93 |
| DBil (umol/L) | 0.997(0.994,1.000) | 0.040 | 304.58 | 0.586(0.511,0.662) | 269.15 | 0.36 | 0.86 |
| IBil (umol/L) | 0.993(0.986,0.999) | 0.025 | 303.86 | 0.572(0.496,0.648) | 121.10 | 0.28 | 0.87 |
| ALT (U/L) | 1.002(1.001,1.003) | <0.0001 | 288.30 | 0.650(0.578,0.722) | 268.50 | 0.46 | 0.78 |
| AST (U/L) | 1.001(1.000,1.002)) | 0.008 | 301.61 | 0.626(0.552,0.699) | 164.00 | 0.75 | 0.49 |
| m-AST (U/L) | 1.010(1.003,1.017) | 0.006 | 299.06 | 0.647(0.574,0.719) | 16.45 | 0.80 | 0.46 |
| CHE (U/L) | 1.000(1.000,1.001) | <0.0001 | 295.92 | 0.628(0.554,0.701) | 30077.50 | 0.63 | 0.62 |
| CR (umol/L) | 0.993(0.987,0.999) | 0.022 | 303.94 | 0.525(0.449,0.602) | 82.50 | 0.29 | 0.85 |
| Meld score | 0.888(0.825,0.956) | 0.002 | 296.33 | 0.645(0.572,0.718) | 27.25 | 0.40 | 0.87 |
| 3-Month Mortality (%) | 0.963(0.941,0.985) | 0.001 | 297.95 | 0.558(0.482,0.634) | 36.30 | 0.23 | 0.94 |

Abbreviations: OR, odds ratio; QIC, Quasi likelihood under the independence model criterion; AUC, area under the curve; PTA, prothrombin time activity; INR, international normalized ratio; APTT, activated partial thromboplastin time; TBil, total bilirubin; DBIL, direct bilirubin; IBil, indirect bilirubin; ALT, alanine aminotransferase; AST, aspartate aminotransferase; m-AST, mitochondrial aspartate transaminase; CHE, cholinesterase; CR, creatinine; MELD, model for end stage liver disease; 3-Month Mortality, 3-month mortality based on MELD scores;

**Supplementary table 2.** Relationships between laboratory biomarkers before ALSS treatment and in-hospital outcomes

| Parameters | Survival in-hospital | |  |  |  |
| --- | --- | --- | --- | --- | --- |
|  | YES | NO | Wald Chi-Square | *P* | QIC |
|  | (N=363) | (N=363) |  |  |  |
| PTA (%) | 47.00(36.00,55.00) | 36.00(29.00,48.00) | 15.91 | <0.0001 | 968.98 |
| INR | 1.71(1.49,2.09) | 2.03(1.57,2.44) | 7.59 | 0.006 | 992.46 |
| APTT (S) | 41.89±8.22 | 48.73±10.82 | 22.63 | <0.0001 | 931.07 |
| FBG (s) | 19.00(14.60,26.2) | 19.00(14.70,25.80) | 0.32 | 0.572 | 1017.23 |
| TT (s) | 19.66±5.20 | 20.43±4.27 | 1.49 | 0.222 | 1015.85 |
| AT (%) | 43.50(34.00,54.00) | 35.00(28.00,42.00) | 8.16 | 0.004 | 993.10 |
| D-dimer (mg/L,DDU) | 1.20(0.45,2.38) | 2.14(1.18,3.10) | 7.63 | 0.006 | 993.41 |
| FDP (mg/L) | 4.40(2.20,9.40) | 6.05(3.75,8.10) | 0.03 | 0.87 | 1019.77 |
| TP (g/L) | 55.56±8.14 | 51.33±6.91 | 22.25 | <0.0001 | 959.10 |
| ALB (g/L) | 32.88±4.76 | 33.29±4.40 | 0.87 | 0.35 | 1016.68 |
| Glb (g/L) | 22.25(18.70,25.50) | 17.80(14.30,20.90) | 37.92 | <0.0001 | 922.59 |
| A/G | 1.49(1.26,1.77) | 1.88(1.55,2.39) | 1.76 | 0.184 | 1008.71 |
| TBil (umol/L) | 224.40(165.20,300.90) | 359.20(270.50,475.10) | 117.10 | <0.0001 | 775.75 |
| DBil (umol/L) | 152.80(97.40,207.90) | 248.90(170.90,351.30) | 95.05 | <0.0001 | 813.42 |
| IBil (umol/L) | 77.45±25.91 | 111.96±40.54 | 90.87 | <0.0001 | 846.76 |
| DBil/TBil | 0.67(0.59,0.72) | 0.70(0.63,0.76) | 11.55 | 0.001 | 989.41 |
| ALT (U/L) | 98.00(60.00,221.00) | 80.00(42.00,168.00) | 1.62 | 0.203 | 1015.70 |
| AST (U/L) | 133.00(94.00,229.00) | 114.00(65.00,207.00) | 0.43 | 0.511 | 1018.76 |
| m-AST (U/L) | 15.10(10.40,26.20) | 12.70(6.80,21.60) | 1.21 | 0.271 | 1016.09 |
| m-AST/AST | 0.11(0.09,0.14) | 0.10(0.08,0.15) | 0.02 | 0.897 | 1000.29 |
| ALP (U/L) | 156.00(115.00,243.75) | 153.50(114.75,201.25) | 0.50 | 0.478 | 904.27 |
| GGT (U/L) | 204.00(119.00,353.00) | 137.00(77.00,229.00) | 8.23 | 0.004 | 976.79 |
| TBA (umol/L) | 367.80(263.30,470.10) | 388.15(270.83,489.10) | 0.29 | 0.589 | 980.19 |
| CHE (U/L) | 3291.00(2646.00,3935.00) | 2812.00(2255.00,3606.00) | 12.51 | <0.0001 | 992.72 |
| PA (mg/dl) | 7.55(5.50,9.60) | 5.30(4.00,6.90) | 14.17 | <0.0001 | 967.03 |
| CR (umol/L) | 65.00(55.00,79.00) | 67.00(55.00,86.00) | 4.59 | 0.032 | 1008.24 |
| Na (mmol/L) | 137.00(134.30,139.30) | 135.70(132.40,138.30) | 7.60 | 0.006 | 1000.23 |
| Urea (mmol/L) | 5.17(4.19,6.77) | 6.27(4.47,8.70) | 8.50 | 0.004 | 996.92 |
| UA (umol/L) | 148.00(122.00,206.00) | 183.00(145.50,242.00) | 9.14 | 0.002 | 998.12 |
| NEUT (%) | 72.20(67.30,78.15) | 74.95(68.85,80.70) | 7.38 | 0.007 | 1004.79 |
| NLR | 4.24(2.86,6.75) | 5.60(3.13,10.38) | 0.32 | 0.573 | 524.24 |
| PLR | 104.55(66.13,159.38) | 110.96(68.35,163.37) | 0.58 | 0.446 | 516.99 |
| PLT (10^9^/L) | 124.00(92.00,149.00) | 84.00(57.50,116.00) | 16.00 | <0.0001 | 981.75 |
| Meld score | 24.15±4.03 | 28.05±4.61 | 66.46 | <0.0001 | 881.50 |
| 3-Month Mortality (%) | 19.60(19.60,19.60) | 19.60(19.60,52.60) | 59.85 | <0.0001 | 931.65 |

Abbreviations: QIC, Quasi likelihood under the independence model criterion; PTA, prothrombin time activity; INR, international normalized ratio; APTT, activated partial thromboplastin time; FBG, fibrinogen; TT, thrombin time; AT, antithrombin; FDP, fibrinogen degradation products; TP, total protein; ALB albumin; Glb, globulin; TBil, total bilirubin; DBIL, direct bilirubin; IBil, indirect bilirubin; ALT, alanine aminotransferase; AST, aspartate aminotransferase; m-AST, mitochondrial aspartate transaminase; ALP, alkaline phosphatase; GGT, gamma-glutamyl transpeptidase; TBA, total bile acid; CHE, cholinesterase; PA, prealbumin; CR, creatinine; UA, uric acid; NEUT, neutrophils; NLR, neutrophil-to-lymphocyte ratio; PLR, platelet-to-lymphocyte ratio; PLT, platelets; MELD, model for end stage liver disease; 3-Month Mortality, 3-month mortality based on MELD scores;

**Supplementary table 3.** Univariate GEE model for patients with HBV-ACLF with full dataset for mortality before ALSS treatment

| Predictors | OR (95% CI) | *P* | QIC | AUC (95% CI) | Cut-off | Sensitivity | Specificity |
| --- | --- | --- | --- | --- | --- | --- | --- |
| PTA (%) | 1.037(1.019,1.056) | <0.0001 | 968.98 | 0.652(0.612,0.692) | 40.50 | 0.68 | 0.62 |
| INR | 0.62(0.441,0.871) | 0.006 | 992.46 | 0.617(0.576,0.658) | 1.93 | 0.56 | 0.69 |
| APTT (S) | 0.923(0.894,0.954) | <0.0001 | 931.07 | 0.709(0.672,0.747) | 43.55 | 0.69 | 0.64 |
| AT (%) | 1.019(1.006,1.032) | 0.004 | 993.10 | 0.654(0.613,0.694) | 39.25 | 0.62 | 0.73 |
| D-dimer (mg/L,DDU) | 0.813(0.703,0.942) | 0.006 | 993.41 | 0.656(0.616,0.696) | 1.48 | 0.70 | 0.60 |
| TP (g/L) | 1.088(1.051,1.026) | <0.0001 | 959.10 | 0.673(0.634,0.712) | 54.85 | 0.55 | 0.75 |
| Glb (g/L) | 1.138(1.092,1.186) | <0.0001 | 922.59 | 0.716(0.678,0.753) | 20.75 | 0.64 | 0.74 |
| TBil (umol/L) | 0.989(0.987,0.991) | <0.0001 | 775.75 | 0.808(0.777,0.839) | 332.15 | 0.58 | 0.85 |
| DBil (umol/L) | 0.988(0.985,0.99) | <0.0001 | 813.42 | 0.782(0.749,0.814) | 239.55 | 0.55 | 0.86 |
| IBil (umol/L) | 0.97(0.963,0.976) | <0.0001 | 846.76 | 0.762(0.728,0.797) | 107.75 | 0.52 | 0.88 |
| DBil/TBil | 0.013(0.001,0.162) | 0.001 | 989.41 | 0.614(0.573,0.654) | 0.74 | 0.36 | 0.81 |
| GGT (U/L) | 1.002(1.001,1.003) | 0.004 | 976.79 | 0.658(0.619,0.697) | 185.50 | 0.56 | 0.68 |
| CHE (U/L) | 1.000(1.000,1.001) | <0.0001 | 992.72 | 0.613(0.572,0.654) | 2605.50 | 0.78 | 0.44 |
| PA (mg/dl) | 1.159(1.073,1.251) | <0.0001 | 967.03 | 0.700(0.661,0.738) | 6.55 | 0.64 | 0.73 |
| CR (umol/L) | 0.993(0.987,0.999) | 0.032 | 1008.24 | 0.534(0.492,0.576) | 85.50 | 0.25 | 0.85 |
| Na (mmol/L) | 1.066(1.019,1.116) | 0.006 | 1000.23 | 0.599(0.558,0.641) | 135.98 | 0.65 | 0.55 |
| Urea (mmol/L) | 0.908(0.852,0.969) | 0.004 | 996.92 | 0.597(0.556,0.639) | 6.21 | 0.51 | 0.69 |
| UA (umol/L) | 0.997(0.994,0.999) | 0.002 | 998.12 | 0.614(0.573,0.656) | 150.50 | 0.73 | 0.53 |
| NEUT (%) | 0.97(0.949,0.992) | 0.007 | 1004.79 | 0.580(0.538,0.621) | 72.95 | 0.63 | 0.57 |
| PLT (10^9^/L) | 1.007(1.004,1.011) | <0.0001 | 981.75 | 0.665(0.625,0.705) | 90.50 | 0.76 | 0.58 |
| Meld score | 0.808(0.768,0.850) | <0.0001 | 881.50 | 0.744(0.708,0.779) | 26.25 | 0.61 | 0.76 |
| 3-Month Mortality(%) | 0.95(0.937,0.962) | <0.0001 | 931.65 | 0.657(0.617,0.696) | 27.30 | 0.35 | 0.91 |

Abbreviations: OR, odds ratio; QIC, Quasi likelihood under the independence model criterion; AUC, area under the curve; PTA, prothrombin time activity; INR, international normalized ratio; APTT, activated partial thromboplastin time; AT, antithrombin; TP, total protein; Glb, globulin; TBil, total bilirubin; DBIL, direct bilirubin; IBil, indirect bilirubin; GGT, gamma-glutamyl transpeptidase; CHE, cholinesterase; PA, prealbumin; CR, creatinine; UA, uric acid; NEUT, neutrophils; PLT, platelets; MELD, model for end stage liver disease; 3-Month Mortality, 3-month mortality based on MELD scores;

**Supplementary table 4.** Relationships between laboratory biomarkers after ALSS treatment and in-hospital outcomes

| Parameters | Survival in-hospital | |  |  |  |
| --- | --- | --- | --- | --- | --- |
|  | YES | NO | Wald Chi-Square | *P* | QIC |
|  | (N=363) | (N=363) |  |  |  |
| PTA (%) | 70.00(58.00,84.00) | 49.00(41.00,70.00) | 10.48 | 0.001 | 943.50 |
| INR | 1.26(1.11,1.44) | 1.63(1.27,1.93) | 26.93 | <0.0001 | 877.39 |
| APTT (S) | 40.10(34.70,44.70) | 44.20(39.00,51.00) | 11.99 | 0.001 | 959.31 |
| FBG (s) | 19.10(14.70,24.80) | 18.00(14.30,23.00) | 2.00 | 0.158 | 1014.78 |
| TT (s) | 19.38±4.02 | 19.92±5.23 | 1.03 | 0.311 | 1016.91 |
| AT (%) | 48.00(38.00,64.50) | 39.00(27.50,56.00) | 2.10 | 0.147 | 665.89 |
| D-dimer (mg/L,DDU) | 1.10(0.51,1.90) | 1.86(1.10,3.03) | 5.66 | 0.017 | 989.92 |
| FDP (mg/L) | 3.90(2.10,7.90) | 6.00(3.23,9.60) | 1.45 | 0.229 | 630.31 |
| TP (g/L) | 55.55±8.07 | 48.60±6.18 | 61.95 | <0.0001 | 844.92 |
| ALB (g/L) | 33.55±5.32 | 33.15±4.51 | 0.62 | 0.432 | 778.43 |
| Glb (g/L) | 21.15(18.30,24.50) | 15.15(12.60,19.50) | 63.64 | <0.0001 | 820.60 |
| A/G | 1.60(1.32,1.94) | 2.09(1.60,2.70) | 0.67 | 0.412 | 802.12 |
| TBil (umol/L) | 139.40(96.50,185.20) | 220.10(172.20,277.10) | 76.26 | <0.0001 | 798.19 |
| DBil (umol/L) | 80.10(52.90,121.00) | 137.70(100.60,197.40) | 72.15 | <0.0001 | 831.45 |
| IBil (umol/L) | 54.80(42.50,69.00) | 77.20(58.10,96.20) | 47.75 | <0.0001 | 880.67 |
| DBil/TBil | 0.59±0.10 | 0.64±0.12 | 17.16 | <0.0001 | 978.75 |
| ALT (U/L) | 74.00(38.00,150.50) | 63.00(35.00,159.50) | 0.61 | 0.436 | 785.26 |
| AST (U/L) | 96.00(64.00,157.00) | 100.50(56.00,183.50) | 2.64 | 0.104 | 779.09 |
| m-AST (U/L) | 11.50(8.10,18.20) | 11.90(5.90,20.00) | 1.01 | 0.315 | 781.21 |
| m-AST/AST | 0.12(0.09,0.15) | 0.10(0.08,0.15) | 0.37 | 0.542 | 776.81 |
| ALP (U/L) | 153.00(109.00,225.50) | 152.00(114.00,197.50) | 0.03 | 0.872 | 786.61 |
| GGT (U/L) | 192.00(113.00,305.75) | 133.00(73.00,208.00) | 7.28 | 0.007 | 992.64 |
| TBA (umol/L) | 271.30(140.90,360.90) | 290.80(212.20,359.60) | 2.69 | 0.101 | 1013.56 |
| CHE (U/L) | 2953.50(2170.00,3907.50) | 2929.00(2174.00,3872.00) | 0.49 | 0.485 | 672.47 |
| PA (mg/dl) | 8.30(7.10,10.80) | 5.30(4.00,7.10) | 22.82 | <0.0001 | 886.88 |
| CR (umol/L) | 64.00(54.00,74.00) | 74.00(61.00,96.00) | 16.35 | <0.0001 | 960.38 |
| Na (mmol/L) | 137.80(134.80,139.90) | 136.10(132.30,138.70) | 52.28 | <0.0001 | 1012.86 |
| Urea (mmol/L) | 5.50(4.37,7.10) | 7.47(4.98,12.53) | 23.58 | <0.0001 | 950.87 |
| UA (umol/L) | 144.50(125.00,196.00) | 205.00(149.75,272.00) | 11.32 | 0.001 | 967.08 |
| NEUT (%) | 70.90(64.90,78.90) | 76.65(71.40,81.90) | 41.87 | <0.0001 | 953.92 |
| NLR | 4.04(3.20,5.57) | 7.90(5.12,14.09) | 28.68 | <0.0001 | 878.12 |
| PLR | 96.05(61.36,152.51) | 92.86(63.44,163.43) | 1.39 | 0.239 | 406.26 |
| PLT(10^9^/L) | 119.00(93.00,159.00) | 71.50(52.00,104.00) | 25.09 | <0.0001 | 935.59 |
| Meld score | 18.00(16.00,21.00) | 24.00(21.00,27.00) | 71.24 | <0.0001 | 715.25 |
| 3-Month Mortality (%) | 6.00(6.00,19.60) | 19.60(19.60,19.60) | 70.52 | <0.0001 | 768.34 |

Abbreviations: QIC, Quasi likelihood under the independence model criterion; PTA, prothrombin time activity; INR, international normalized ratio; APTT, activated partial thromboplastin time; FBG, fibrinogen; TT, thrombin time; AT, antithrombin; FDP, fibrinogen degradation products; TP, total protein; ALB albumin; Glb, globulin; TBil, total bilirubin; DBIL, direct bilirubin; IBil, indirect bilirubin; ALT, alanine aminotransferase; AST, aspartate aminotransferase; m-AST, mitochondrial aspartate transaminase; ALP, alkaline phosphatase; GGT, gamma-glutamyl transpeptidase; TBA, total bile acid; CHE, cholinesterase; PA, prealbumin; CR, creatinine; UA, uric acid; NEUT, neutrophils; NLR, neutrophil-to-lymphocyte ratio; PLR, platelet-to-lymphocyte ratio; PLT, platelets; MELD, model for end stage liver disease; 3-Month Mortality, 3-month mortality based on MELD scores;

**Supplementary table 5.** Univariate GEE model for patients with HBV-ACLF with full dataset for mortality after ALSS treatment

| Predictors | OR (95% CI) | *P* | QIC | AUC (95% CI) | Cut-off | Sensitivity | Specificity |
| --- | --- | --- | --- | --- | --- | --- | --- |
| PTA (%) | 1.030(1.012,1.048) | 0.001 | 943.50 | 0.733(0.695,0.73) | 50.50 | 0.91 | 0.54 |
| INR | 0.074(0.028,0.198) | <0.0001 | 877.39 | 0.733(0.695,0.77) | 1.61 | 0.53 | 0.90 |
| APTT (s) | 0.939(0.907,0.973) | 0.001 | 959.31 | 0.671(0.632,0.710) | 42.95 | 0.56 | 0.70 |
| D-dimer (mg/L,DDU) | 0.768(0.617,0.954) | 0.017 | 989.92 | 0.678(0.639,0.717) | 1.40 | 0.70 | 0.61 |
| TP (g/L) | 1.174(1.128,1.221) | <0.0001 | 844.92 | 0.776(0.742,0.81) | 51.95 | 0.74 | 0.71 |
| Glb(g/L) | 1.246(1.180,1.315) | <0.0001 | 820.60 | 0.797(0.765,0.83) | 19.85 | 0.68 | 0.83 |
| TBil (umol/L) | 0.983(0.980,0.987) | <0.0001 | 798.19 | 0.803(0.771,0.834) | 164.05 | 0.82 | 0.66 |
| DBil (umol/L) | 0.982(0.977,0.986) | <0.0001 | 831.45 | 0.778(0.745,0.811) | 92.25 | 0.79 | 0.63 |
| IBil (umol/L) | 0.964(0.953,0.974) | <0.0001 | 880.67 | 0.740(0.705,0.776) | 62.35 | 0.70 | 0.68 |
| DBil/TBil | 0.013(0.002,0.100) | <0.0001 | 978.75 | 0.632(0.592,0.672) | 0.66 | 0.45 | 0.75 |
| GGT (U/L) | 1.001(1.000,1.002) | 0.007 | 992.64 | 0.648(0.608,0.687) | 145.50 | 0.66 | 0.58 |
| PA (mg/dl) | 1.318(1.177,1.475) | <0.0001 | 886.88 | 0.784(0.751,0.818) | 6.65 | 0.79 | 0.73 |
| CR (umol/L) | 0.979(0.969,0.989) | <0.0001 | 960.38 | 0.659(0.620,0.699) | 87.50 | 0.39 | 0.89 |
| Na (mmol/L) | 1.000(1.000,1.000) | <0.0001 | 1012.86 | 0.605(0.564,0.646) | 137.25 | 0.56 | 0.63 |
| Urea (mmol/L) | 0.849(0.795,0.907) | <0.0001 | 950.87 | 0.657(0.617,0.697) | 6.58 | 0.60 | 0.71 |
| UA (umol/L) | 0.995(0.992,0.998) | 0.001 | 967.08 | 0.659(0.618,0.70) | 172.50 | 0.64 | 0.70 |
| NEUT (%) | 0.941(0.924,0.958) | <0.0001 | 953.92 | 0.66(0.621,0.70) | 72.30 | 0.75 | 0.55 |
| NLR | 0.803(0.741,0.870) | <0.0001 | 878.12 | 0.748(0.711,0.785) | 6.78 | 0.66 | 0.85 |
| PLT (10^9^/L) | 1.012(1.007,1.017) | <0.0001 | 935.59 | 0.74(0.703,0.777) | 86.50 | 0.80 | 0.66 |
| Meld score | 0.695(0.639,0.756) | <0.0001 | 715.25 | 0.851(0.823,0.878) | 22.25 | 0.69 | 0.88 |
| 3-Month Mortality(%) | 0.849(0.817,0.882) | <0.0001 | 768.34 | 0.79(0.757,0.823) | 12.65 | 0.89 | 0.64 |

Abbreviations: OR, odds ratio; QIC, Quasi likelihood under the independence model criterion; AUC, area under the curve; PTA, prothrombin time activity; INR, international normalized ratio; APTT, activated partial thromboplastin time; TP, total protein; Glb, globulin; TBil, total bilirubin; DBIL, direct bilirubin; IBil, indirect bilirubin; GGT, gamma-glutamyl transpeptidase; PA, prealbumin; CR, creatinine; UA, uric acid; NEUT, neutrophils; NLR, neutrophil-to-lymphocyte ratio; PLT, platelets; MELD, model for end stage liver disease; 3-Month Mortality, 3-month mortality based on MELD scores;

**Supplementary table 6.** Relationships between change ratio (change ratio = (Post-ALSS-Pre-ALSS)/Pre-ALSS)) of each laboratory biomarker and in-hospital outcomes

| Parameters | Survival in-hospital | |  |  |  |
| --- | --- | --- | --- | --- | --- |
|  | YES | NO | Wald Chi-Square | *P* | QIC |
|  | (N=363) | (N=363) |  |  |  |
| PTA (%) | 0.52(0.25,1.02) | 0.41(0.16,0.78) | 1.78 | 0.040 | 1008.25 |
| INR | -0.26(-0.40,-0.14) | -0.23(-0.35,-0.09) | 9.19 | 0.002 | 1000.91 |
| APTT (s) | -0.02(-0.09,-0.05) | -0.05(-0.14,-0.06) | 0.00 | 0.951 | 1016.74 |
| FBG (s) | -0.10(-0.24,0.21) | -0.11(-0.29,0.19) | 1.93 | 0.165 | 1014.90 |
| TT (s) | 0.00(-0.06,0.07) | -0.04(-0.11,0.01) | 0.73 | 0.394 | 1016.53 |
| AT (%) | 0.12(-0.04,0.29) | 0.18(-0.11,0.38) | 3.61 | 0.058 | 1013.32 |
| D-dimer (mg/L,DDU) | 0.00(-0.31,0.33) | -0.03(-0.43,0.63) | 6.11 | 0.013 | 1007.94 |
| FDP (mg/L) | -0.03(-0.4,0.22) | 0.00(-0.32,0.35) | 1.80 | 0.180 | 1014.84 |
| TP (g/L) | -0.00(-0.08,0.07) | -0.05(-0.11,0.02) | 42.97 | <0.0001 | 986.63 |
| ALB (g/L) | 0.02(-0.06,0.11) | 0.02(-0.09,0.07) | 21.87 | <0.0001 | 1005.76 |
| Glb (g/L) | -0.05(-0.16,0.10) | -0.13(-0.28,0.05) | 16.08 | <0.0001 | 1003.65 |
| A/G | 0.05(-0.10,0.22) | 0.15(-0.07,0.42) | 0.00 | 0.984 | 1016.70 |
| TBil (umol/L) | -0.38(-0.44,-0.28) | -0.39(-0.47,-0.28) | 1.17 | 0.279 | 1015.56 |
| DBil (umol/L) | -0.44(-0.51,-0.34) | -0.43(-0.52,-0.31) | 2.39 | 0.122 | 1014.06 |
| IBil (umol/L) | -0.26(-0.38,-0.14) | -0.28(-0.42,-0.09) | 1.26 | 0.261 | 1014.80 |
| DBil/TBil | -0.10(-0.16,-0.03) | -0.08(-0.16,0.01) | 4.38 | 0.036 | 1011.23 |
| ALT (U/L) | -0.26(-0.55,0.03) | -0.10(-0.46,0.38) | 12.91 | <0.0001 | 995.40 |
| AST (U/L) | -0.28(-0.48,-0.06) | -0.08(-0.46,0.44) | 9.49 | 0.002 | 987.11 |
| m-AST (U/L) | -0.25(-0.47,-0.02) | -0.08(-0.45,0.56) | 3.99 | 0.046 | 995.73 |
| m-AST/AST | 0.01(-0.18,0.30) | 0.04(-0.33,0.43) | 18.00 | <0.0001 | 1006.29 |
| ALP (U/L) | -0.07(-0.25,0.20) | 0.02(-0.26,0.30) | 1.49 | 0.222 | 1015.30 |
| GGT (U/L) | -0.09(-0.32,0.25) | -0.07(-0.36,0.45) | 10.09 | 0.001 | 1006.97 |
| TBA (umol/L) | -0.23 (-0.44,-0.06) | -0.25(-0.38,-0.03) | 1.24 | 0.265 | 1014.22 |
| CHE (U/L) | -0.09(-0.22,0.10) | -0.03(-0.23,0.30) | 0.78 | 0.377 | 1017.78 |
| PA (mg/dl) | 0.13(-0.02,0.43) | 0.00(-0.27,0.42) | 3.74 | 0.053 | 1013.29 |
| CR (umol/L) | -0.06(-0.17,0.10) | 0.11(-0.14,0.46) | 69.58 | <0.0001 | 968.48 |
| Na (mmol/L) | 0.00(-0.02,0.02) | 0.00(-0.02,0.03) | 2.60 | 0.107 | 1014.39 |
| Urea (mmol/L) | 0.02(-0.15,0.26) | 0.14(-0.18,0.69) | 37.68 | <0.0001 | 989.03 |
| UA (umol/L) | -0.04(-0.22,0.25) | 0.05(-0.25,0.47) | 0.00 | 0.973 | 1016.56 |
| NEUT (%) | -0.01(-0.11,0.07) | 0.03(-0.04,0.10) | 7.19 | 0.007 | 1008.37 |
| NLR | -0.09(-0.38,0.36) | 0.33(-0.21,1.08) | 0.65 | 0.419 | 1024.31 |
| PLR | -0.05 (-0.35,0.34) | -0.14(-0.47,0.26) | 2.31 | 0.129 | 1014.65 |
| PLT (10^9^/L) | 0.00(-0.29,0.39) | -0.13(-0.37,0.14) | 4.28 | 0.039 | 1012.59 |
| Meld score | -0.23(-0.32,-0.14) | -0.14(-0.23,-0.04) | 60.99 | <0.0001 | 932.79 |
| 3-Month Mortality (%) | -0.69(-0.69,0.00) | 0.00(-0.63,0.00) | 30.97 | <0.0001 | 955.98 |

Abbreviations: QIC, Quasi likelihood under the independence model criterion; PTA, prothrombin time activity; INR, international normalized ratio; APTT, activated partial thromboplastin time; FBG, fibrinogen; TT, thrombin time; AT, antithrombin; FDP, fibrinogen degradation products; TP, total protein; ALB albumin; Glb, globulin; TBil, total bilirubin; DBIL, direct bilirubin; IBil, indirect bilirubin; ALT, alanine aminotransferase; AST, aspartate aminotransferase; m-AST, mitochondrial aspartate transaminase; ALP, alkaline phosphatase; GGT, gamma-glutamyl transpeptidase; TBA, total bile acid; CHE, cholinesterase; PA, prealbumin; CR, creatinine; UA, uric acid; NEUT, neutrophils; NLR, neutrophil-to-lymphocyte ratio; PLR, platelet-to-lymphocyte ratio; PLT, platelets; MELD, model for end stage liver disease; 3-Month Mortality, 3-month mortality based on MELD scores;

**Supplementary table 7.** Univariate GEE model for patients with HBV-ACLF with full change ratio (Post-ALSS-Pre-ALSS)/Pre-ALSS) dataset for mortality

| Predictors | OR (95% CI) | *P* | QIC | AUC (95% CI) | Cut-off | Sensitivity | Specificity |
| --- | --- | --- | --- | --- | --- | --- | --- |
| PTA (%) | 1.528(1.020,2.287) | 0.040 | 1008.25 | 0.579(0.538,0.621) | 0.34 | 0.67 | 0.46 |
| INR | 0.194(0.067,0.560) | 0.002 | 1000.91 | 0.575(0.534,0.617) | -0.12 | 0.31 | 0.82 |
| D-dimer (mg/L,DDU) | 0.916(0.855,0.982) | 0.013 | 1007.94 | 0.503(0.460,0.545) | -0.34 | 0.76 | 0.34 |
| TP (g/L) | 22.233(8.795,56.200) | <0.0001 | 986.63 | 0.613(0.572,0.653) | 0.00 | 0.50 | 0.72 |
| ALB (g/L) | 4.194(2.300,7.649) | <0.0001 | 1005.76 | 0.573(0.532,0.615) | -0.06 | 0.75 | 0.38 |
| Glb (g/L) | 2.366(1.553,3.603) | <0.0001 | 1003.65 | 0.601(0.559,0.642) | -0.17 | 0.77 | 0.46 |
| DBil/TBil | 0.270(0.079,0.921) | 0.036 | 1011.23 | 0.550(0.508,0.592) | -0.02 | 0.33 | 0.80 |
| ALT (U/L) | 0.837(0.759,0.922) | <0.0001 | 995.40 | 0.588(0.546,0.629) | -0.03 | 0.47 | 0.73 |
| AST (U/L) | 0.653(0.498,0.856) | 0.002 | 987.11 | 0.617(0.576,0.658) | 0.04 | 0.44 | 0.82 |
| m-AST (U/L) | 0.726(0.530,0.994) | 0.046 | 995.73 | 0.607(0.566,0.649) | -0.05 | 0.48 | 0.74 |
| m-AST/AST | 0.76(0.669,0.862) | <0.0001 | 1006.29 | 0.501(0.458,0.543) | -0.27 | 0.71 | 0.15 |
| GGT (U/L) | 0.919(0.872,0.968) | 0.001 | 1006.97 | 0.511(0.469,0.554) | 0.35 | 0.28 | 0.79 |
| CR (umol/L) | 0.263(0.192,0.360) | <0.0001 | 968.48 | 0.622(0.581,0.664) | 0.10 | 0.52 | 0.75 |
| Urea (mmol/L) | 0.569(0.475,0.681) | <0.0001 | 989.03 | 0.564(0.522,0.607) | 0.37 | 0.38 | 0.81 |
| NEUT (%) | 0.326(0.144,0.740) | 0.007 | 1008.37 | 0.592(0.551,0.634) | -0.05 | 0.78 | 0.41 |
| PLT (10^9^/L) | 1.180(1.009,1.380) | 0.039 | 1012.59 | 0.578(0.537,0.620) | -0.04 | 0.54 | 0.64 |
| Meld score | 0.008(0.003,0.028) | <0.0001 | 932.79 | 0.683(0.645,0.722) | -0.18 | 0.63 | 0.66 |
| 3-Month Mortality (%) | 0.251(0.155,0.409) | <0.0001 | 955.98 | 0.688(0.650,0.727) | -0.66 | 0.89 | 0.53 |

Abbreviations: OR, odds ratio; QIC, Quasi likelihood under the independence model criterion; AUC, area under the curve; PTA, prothrombin time activity; INR, international normalized ratio; TP, total protein; ALB albumin; Glb, globulin; ALT, alanine aminotransferase; AST, aspartate aminotransferase; m-AST, mitochondrial aspartate transaminase; GGT, gamma-glutamyl transpeptidase; CR, creatinine; NEUT, neutrophils; PLT, platelets; MELD, model for end stage liver disease; 3-Month Mortality, 3-month mortality based on MELD scores;
